# Phenotypic Analysis of a Family of Transcriptional Regulators, the Zinc Cluster Proteins, in the Human Fungal Pathogen *Candida glabrata*

**DOI:** 10.1534/g3.113.010199

**Published:** 2014-03-21

**Authors:** Natalia Klimova, Ralph Yeung, Nadezda Kachurina, Bernard Turcotte

**Affiliations:** * Department of Medicine, McGill University Health Centre, McGill University, Montréal, Québec, Canada H3A 1A1; † Department of Biochemistry, McGill University Health Centre, McGill University, Montréal, Québec, Canada H3A 1A1; ‡ Department of Microbiology and Immunology, McGill University Health Centre, McGill University, Montréal, Québec, Canada H3A 1A1

**Keywords:** transcriptional regulators, *Candida glabrata*, zinc cluster proteins, phenotypic analysis, drug resistance

## Abstract

*Candida glabrata* is the second most important human fungal pathogen. Despite its formal name, *C. glabrata* is in fact more closely related to the nonpathogenic budding yeast *Saccharomyces cerevisiae*. However, less is known about the biology of this pathogen. Zinc cluster proteins form a large family of transcriptional regulators involved in the regulation of numerous processes such as the control of the metabolism of sugars, amino acids, fatty acids, as well as drug resistance. The *C. glabrata* genome encodes 41 known or putative zinc cluster proteins, and the majority of them are uncharacterized. We have generated a panel of strains carrying individual deletions of zinc cluster genes. Using a novel approach relying on tetracycline for conditional expression in *C. glabrata* at the translational level, we show that only two zinc cluster genes are essential. We have performed phenotypic analysis of nonessential zinc cluster genes. Our results show that two deletion strains are thermosensitive whereas two strains are sensitive to caffeine, an inhibitor of the target of rapamycin pathway. Increased salt tolerance has been observed for eight deletion strains, whereas one strain showed reduced tolerance to salt. We have also identified a number of strains with increased susceptibility to the antifungal drugs fluconazole and ketoconazole. Interestingly, one deletion strain showed decreased susceptibility to the antifungal micafungin. In summary, we have assigned phenotypes to more than half of the zinc cluster genes in *C. glabrata*. Our study provides a resource that will be useful to better understand the biological role of these transcription factors.

The fungal *Candida* species are the fourth most common cause of hospital-acquired infections and rank just after staphylococci and enterococci (Coleman and Mylonakis 2009; Kim and Sudbery 2011). In the recent years, a new emerging trend has been observed with a shift toward infections with species other than *C. albicans* [reviewed in (Miceli *et al.* 2011)]. For example, *C. glabrata* is now the second most important cause of fungal infections in humans (Roetzer *et al.* 2010). Despite its formal name, *C. glabrata* is more closely related to the nonpathogenic baker’s yeast *Saccharomyces cerevisiae*. The *C. glabrata* genome contains 12.3 Mb and approximately 5300 coding genes (Dujon *et al.* 2004). *C. glabrata* has gained genes involved in adhesion to mammalian cells [*e.g.*, *EPA* genes encoding adhesins (Castano *et al.* 2005; Cormack *et al.* 1999; Silva *et al.* 2011)]. Gene loss has occurred in *C. glabrata* compared with *S. cerevisiae*. For example, *C. glabrata* lacks a number of genes for galactose, phosphate, and sulfur metabolism (Dujon *et al.* 2004; Roetzer *et al.* 2010). In contrast to *C. albicans* and *S. cerevisiae*, *C. glabrata* appears to be asexual and strictly haploid. Pseudohyphal growth has been reported for this organism (Csank and Haynes 2000); however, there is no evidence for hyphal formation or secretion of hydrolases that are associated with *C. albicans* virulence. *C. glabrata* can survive in the environment for many months. As a commensal, it is found on mucosal surfaces and, in contrast to *C. albicans*, tissue penetration is rarely observed (Roetzer *et al.* 2010). In addition, this fungus can survive for an extended period of time in phagocytic cells. Little is known about factors involved in *C. glabrata* virulence.

A very important class of transcriptional regulators is composed of zinc cluster proteins (or binuclear cluster) that form a subfamily of zinc finger proteins. Zinc cluster proteins are exclusively found in fungi and amoeba (Clarke *et al.* 2013; MacPherson *et al.* 2006). These proteins possess the well-conserved motif CysX_2_CysX_6_CysX_5-12_CysX_2_CysX_6-8_Cys. The cysteine residues bind to two zinc atoms, which coordinate folding of the domain involved in DNA recognition (Vallee *et al.* 1991). The vast majority of zinc cluster proteins act as transcriptional regulators [reviewed in ref. (MacPherson *et al.* 2006)]. The family of zinc cluster proteins is best characterized in *S. cerevisiae*. The genome of this organism encodes more than 50 known (or putative) zinc cluster proteins (MacPherson *et al.* 2006). The first and best-studied zinc cluster protein is Gal4, a transcriptional activator of genes involved in the catabolism of galactose (Bhat and Murthy 2001). Many other zinc cluster proteins have been characterized; they control a large number of cellular processes such as the metabolism of amino acids, carbon (sugars and nonfermentable carbon sources), pyrimidine, fatty acid, as well as drug resistance (MacPherson *et al.* 2006; Turcotte *et al.* 2011). A number of zinc cluster proteins are positive regulators, but some function as both activators and repressors [*e.g.*, Rds2 (Turcotte *et al.* 2011)], whereas Rdr1 appears to only down-regulate expression of target genes (Hellauer *et al.* 2002).

## Functional domains of zinc cluster proteins

Quite often, the DNA binding domain (comprising the cysteine-rich region) of zinc cluster proteins is located at the N-terminus whereas an acidic activating domain is located at the C-terminus. A region of low homology of about 80 amino acids, termed the middle homology region, is found among many zinc cluster proteins and is located between the DNA binding and activation domains and may be involved in controlling the transcriptional activity of zinc cluster proteins (Schjerling and Holmberg 1996). In many cases, deletion of the region that bridges the DNA binding domain to the activation domain results in constitutive activity of the transcriptional activator (MacPherson *et al.* 2006). Many zinc cluster proteins bind to DNA as homodimers through a coiled-coil dimerization domain located at the C-terminus of the zinc finger but binding as heterodimers or monomers has also been reported (Akache *et al.* 2004; Cahuzac *et al.* 2001; Mamnun *et al.* 2002; Rottensteiner *et al.* 1997).

## Zinc cluster proteins in *C. glabrata*

In *C. glabrata*, only a handful of zinc cluster proteins have been characterized (Table 1). CgPdr1, the homolog of *S. cerevisiae* Pdr1/Pdr3, confers drug resistance by positively controlling the expression of various genes including the ABC transporters *CDR1*, *PDH1*, and *SNQ2* (Vermitsky *et al.* 2006; Vermitsky and Edlind 2004) that act as drug efflux pumps. CgPdr1 is activated by direct binding of various compounds, including azoles that are antifungal drugs (Thakur *et al.* 2008). As observed in *S. cerevisiae*, mutations in the Cg*PDR1* gene result in hyperactivation of the transcription factor, causing increased resistance to various drugs such as azoles and, unexpectedly, increased virulence (Berila *et al.* 2009; Ferrari *et al.* 2009; Tsai *et al.* 2006; Vermitsky *et al.* 2006). There are two functional homologs of *S. cerevisiae* Upc2/Ecm22 and they were named CgUpc2A and CgUpc2B (Nagi *et al.* 2011). CgUpc2A is an activator of ergosterol biosynthetic genes whereas both CgUpc2A and B are positive regulators of the Cg*AUS1* gene encoding a sterol transporter (Nagi *et al.* 2011). Deletion of Cg*UPC2A* (but not *B*) results in sensitivity to azoles in analogy to *S. cerevisiae*, where we reported that a ∆*upc2* strain is sensitive to ketoconazole whereas no effect was observed with a ∆*ecm22* strain (Akache and Turcotte 2002). Cg*STB5* encodes a repressor of the transporter genes *CDR1*, *PDH1*, and *YOR1* (Noble *et al.* 2013). Finally, Cg*CEP3* encodes a centromeric protein and is the functional homolog of *S. cerevisiae CEP3* (Stoyan and Carbon 2004). In this study, we were interested in characterizing the whole family of zinc cluster proteins in *C. glabrata*. Toward this end, we have generated a panel of strains carrying deletions of zinc cluster genes. Results show that two zinc cluster genes are essential. Using our panel of deletion strains of nonessential zinc cluster genes, we performed phenotypic analysis under various conditions. Phenotypes identified in our screen include sensitivity to oxidative stress, increased tolerance to salt stress, and thermosensitivity. In addition, altered susceptibility to antifungal drugs was observed with a number of deletion strains.

**Table 1:** List of known and putative zinc cluster proteins

Name of Zinc Cluster Gene	Génolevures Code/Name of the Gene	*S. cerevisiae* Homolog	*P* Value	Deletion Strain Generated
*CgZCF1*	CAGL0A00451g Cg*PDR1* (ref. Vermitsky *et al.* 2006)	*PDR1*	2.6e-156	Yes
		*PDR3*	1.5e-105	
*CgZCF2*	CAGL0A00583g	No homolog	N/A	Yes
*CgZCF3*	CAGL0A04455g	*SEF1*	1.9e-257	Yes
		*LEU3*	2.3e-21	
*CgZCF4*	CAGL0B03421g	*HAP1*	5.5e-223	Yes
*CgZCF5*	CAGL0C01199g	*UPC2*	7e-183	Not generated; this gene is not essential in another strain background (Nagi *et al.* 2011)
	*CgUPC2A* (ref. Nagi *et al.* 2011)	*ECM22*	2.8e-169	
*CgZCF6*	CAGL0D02904g	*PPR1*	1.6 e-229	Yes
		*STB5*	5.78e-16	
*CgZCF7*	CAGL0D03850g	*RSC3*	1.1e-142	Yes
		*RSC30*	2.9e-29	
*CgZCF8*	CAGL0E05434g	*TEA1*	7.7e-186	Yes
		*CHA4*	9.1e-106	
*CgZCF9*	CAGL0F02519g	*YJL206C*	3.4e-129	Yes
		*ASG1*	1.1e-91	
*CgZCF10*	CAGL0F03025g	*ARO80*	5.8e-151	Yes
*CgZCF11*	CAGL0F05357g	*UME6*	6e-51	Essential gene
		*LYS14*	1.1e-6	
*CgZCF12*	CAGL0F06743g	*DAL81*	3.8e-184	Yes
		*CHA4*	7.9e-7	
*CgZCF13*	CAGL0F07755g	*CEP3*	2.1e-140	Essential gene
	CgCEP3 (ref. Stoyan and Carbon 2004)	*YKL122C*	7.4e-5	
*CgZCF14*	CAGL0F07865g	*UPC2*	7e-183	Yes
	Cg*UPC2B* (ref. Nagi *et al.* 2011)	*ECM22*	2.8e-169	
*CgZCF15*	CAGL0F07909g	*TBS1*	4.1e-111	Yes
		*HAL9*	2.4e-109	
*CgZCF16*	CAGL0F09229g	*YER184C*	5.4e-80	Yes
		*PDR1*	2.8e-29	
*CgZCF17*	CAGL0G08844g	*ASG1*	5.7e-214	Yes
		*YJL206C*	2.4e-92	
*CgZCF18*	CAGL0G09757g	*YLR278C*	2.2e-264	Yes
		*PPR1*	4.4e-10	
*CgZCF19*	CAGL0H00396g	*LEU3*	2.7e-247	Yes
		*SEF1*	9.2e-17	
*CgZCF20*	CAGL0H01507g	*RSC3*	4.2e-99	Yes
		*RSC30*	3.1e-30	
*CgZCF21*	CAGL0H01683g	*URC2*	1.8e-186	Yes
*CgZCF22*	CAGL0H04367g	*WAR1*	4.2e-133	Yes
*CgZCF23*	CAGL0H06875g	*ARG81*	1.1e-106	Yes
*CgZCF24*	CAGL0I02552g	*STB5*	7.9e-203	Yes
	Cg*STB5* (ref. Noble *et al.* 2013)	*YJL206C*	2e-12	
*CgZCF25*	CAGL0I07755g	*HAL9*	8.8e-196	Yes
		*TBS1*	6.5e-184	
*CgZCF26*	CAGL0J07150g	*OAF1*	4.6e-165	Yes
		*PIP2*	1e-147	
*CgZCF27*	CAGL0K05841g	*HAP1*	8.7 e-159	Yes
*CgZCF28*	CAGL0K06985g	*ERT1*	1e-142	Yes
		*GSM1*	6.1e-30	
*CgZCF29*	CAGL0K11902g	*LYS14*	3.2e-240	Yes
*CgZCF30*	CAGL0L01903g	*RGT1*	1.9e-197	Yes
		*EDS1*	4.9e-54	
*CgZCF31*	CAGL0L03377g	*SIP4*	2.6e-91	Yes
		*CAT8*	1.9e-13	
*CgZCF32*	CAGL0L03674g	*GSM1*	1.7e-81	Yes
		*RDS2*	1.3e-15	
*CgZCF33*	CAGL0L04400g	*YRR1*	9.9e-115	Yes
		*YRM1*	2.1e-112	
*CgZCF34*	CAGL0L04576g	*YRM1*	2.3e-134	Yes
		*YRR1*	2.2e-122	
*CgZCF35*	CAGL0M11440g	*CHA4*	7.8e-171	Yes
		*TEA1*	3e-94	
*CgZCF36*	CAGL0L09383g	*SUT1*	3.3e-33	Yes
		*SUT2*	4.5e-28	
*CgZCF37*	CAGL0L09691g	*PUT3*	2.1e-200	Yes
		*ASG1*	6.8e-20	
*CgZCF38*	CAGL0M12298g	*OAF1*	1.2e-265	Yes
		*PIP2*	2.1e-183	
*CgZCF39*	CAGL0M02651g	*RDS2*	1e-126	Yes
		*ERT1*	5.3e-28	
*CgZCF40*	CAGL0M05907g	*OAF3*	2.6e-116	Yes
*CgZCF41*	CAGL0M03025g	*CAT8*	1.9e-148	Not studied
		*ASG1*	4.2e-13	

*C. glabrata* zinc cluster genes are numbered 1−41. Systematic names (Génolevures code, www.genolevures.org) are also given as well as their gene names (if available). The *S. cerevisiae* closest homologs are also listed along with *P*-values. More information about *S. cerevisiae* zinc cluster genes can be obtained at www.yeastgenome.org. Essential genes are also indicated. Deletion of Cg*ZCF5* was not obtained in the reference strain used in this study.

## Material and Methods

### Strains and media

The wild-type *S. cerevisiae* strain used for construction of plasmids by homologous recombination is BY4741 (*MAT*a *his3Δ1 leu2*Δ*0 met15*Δ*0 ura3*Δ*0*) (Brachmann *et al.* 1998). The wild-type *C. glabrata* strain 66032*ura3* (Vermitsky *et al.* 2006) used to generate the zinc cluster gene deletions is a tight 5-fluoroorotic acid selected *ura3* derivative of strain ATCC 66032. Yeast cells were grown in YPD (2% yeast extract, 1% peptone, 2% glucose) medium or in SD complete medium lacking appropriate auxotrophic components (Adams *et al.* 1997). For selection with the dominant SAT1 marker (Reuss *et al.* 2004), YPD agar plates containing nourseothricin (cloneNAT, Werner BioAgents) at 200 μg/mL were used.

### Plasmids for gene deletion

The overall strategy used to construct plasmids for deletion of zinc genes is schematically shown in Figure 2, and oligonucleotides used to generate plasmids for gene deletion are listed in Supporting Information, Table S1. Plasmid pRS316 (Sikorski and Hieter 1989) was used as a template to amplify the *URA3* marker with oligonucleotides URA3REC-1 and URA3REC-2 that contain sequences homologous to DNA flanking the *Sma*I site in plasmid pRS423 (Sikorski and Hieter 1989). The polymerase chain reaction (PCR) product was transformed into *S. cerevisiae* along with plasmid pRS423 (Brachmann *et al.* 1998) linearized with *Sma*I, and transformants were selected on minimal plates lacking histidine followed by selection on plates lacking uracil. Yeast DNA was isolated according to Hoffman and Winston (1987), and plasmids were recovered by transformation into *Escherichia coli* (DH5α-E) using ElectroMAX electrocompetent cells (Invitrogen) to yield plasmid pRS-URA3. To generate a panel of deletion *C. glabrata* strains (Table 1), a set of plasmids containing disruption cassettes was generated.

The 5′ and 3′ regions flanking of the open reading frames (ORFs) of the zinc cluster genes were amplified by PCR using genomic DNA isolated from strain 66032*ura3*. Oligonucleotides were designed to contain *Sma*I restriction sites at the 5′ and 3′ ends and sequences complementary to the 5′ and 3′ end of the *URA3* marker in pRS-URA3. The 5′ flanking PCR fragment (termed CgZCXXA, where XX refers to a numbered zinc cluster protein) was homologous to the 5′ end of the *URA3* marker in pRS-URA3 and was obtained using primer oligonucleotides CgZCXX-a and CgZCXX-b. Similarly, the resulting 3′ flanking PCR fragment (termed CgZCXXB) was homologous to the 3′ end of the *URA3* marker in pRS-URA3, using primer oligonucleotides CgZCXX-c and CgZCXX-d resulting in PCR products that were approximately 500-bp long. Plasmid pRS-URA3 linearized with *Sma*I was transformed with the 5′ and 3′ PCR products in the *S. cerevisiae* strain BY4741. The flanking PCR fragments were recombined into the *Sma*I-digested pRS-URA3 to generate plasmids via a quadruple recombination. Selection was performed on SD agar plates lacking histidine followed by selection on SD plates lacking uracil. Plasmids were recovered as described previously. Plasmids were named pCg*ZCF1* to *40* (Table 1). Independent clones were verified by DNA sequencing.

### Deletion of zinc cluster genes

Plasmids for deletion of zinc cluster genes were digested with *Sma*I, purified using a QIAquick PCR purification kit (QIAGEN), and 1 µg of plasmid DNA was transformed into the strain 66023*ura3* using the lithium acetate procedure (Gietz *et al.* 1992) except that dimethyl sulfoxide (10% final concentration) was added before the heat shock (42°, 5 min). Cells were plated on SD agar plates lacking uracil, and colonies were restreaked on SD agar plates lacking uracil. Proper integration of the *S. cerevisiae URA3* marker was verified using a reverse PCR primer that overlapped the *URA3* marker (either URA3-CHECK or URA3-CHECK#2) and forward PCR primer that was complementary to genomic sequences upstream of the 5′ region used to perform homologous recombination (termed CgZCXX-check, see Table S1). PCR primers specific to the DNA binding domain for zinc cluster genes were used to ensure complete removal of the ORF of a zinc cluster gene (data not shown). In addition to *URA3*, deletion of Cg*ZCF6* was also obtained using the dominant marker SAT1 (Reuss *et al.* 2004). The SAT1 marker was amplified using oligonucleotides CgZCF6-SAT1F-I and CgZCF6-SAT1R-I and plasmid pSFS2A (Reuss *et al.* 2004). To extend the length of sequences homologous to Cg*ZCF6*, the PCR product was used as a template for a second PCR amplification using oligonucleotides CgZCF6-SAT1F-II and CgZCF6-SAT1R-II. A cassette for deletion of Cg*ZCF23* was obtained by amplifying the Myc-URA3-Myc sequences of plasmid pMPY-3xMyc (Schneider *et al.* 1995) using oligos PET-CgZC23-1 and KO-CgZC23-2 followed by a second amplification using oligonucleotides PET-CgZC23-4 and KO-CgZC23-4. Similarly, one deletion strain for Cg*PDR1* was generated using plasmid pMPY-3xMyc and the oligonucleotides PET-CgZC1-1, KO-CgZC1-2, PET-CgZC1-3, and KO-CgZC1-4.

### Complementation assays

Zinc cluster genes were amplified using the Expand Long Template PCR System (Roche) with genomic DNA isolated from strain 66032*ura3* and oligonucleotides listed in Table S1. Oligonucleotides were designed so that approximately 200−400 bp of sequences flanking an ORF of interest were part of the PCR product. DNA was purified with a QIAquick PCR Purification Kit (QIAGEN) and used to transform deletion strains carrying the *URA3* marker. Cells were then directly plated on FOA plates to select for Ura^−^ cells. With the exception of Cg*ZCF9*, at least two complementation strains (usually three or more strains) for each deletion strain were tested for reversion of the phenotype. All complementation strains tested showed wild-type phenotypes.

### Conditional expression of zinc cluster proteins

The G418^R^ marker of plasmid pADH1-tc3-3XHA (Kotter *et al.* 2009) was replaced by the *S. cerevisiae URA3* marker. To this end, oligonucleotides ScURA3-1 and ScURA3-2 were used to amplify the *URA3* gene using plasmid pRS316 as a template (Sikorski and Hieter 1989). The PCR product was cut with *Bam*HI and *Sac*I and subcloned into pADH1-tc3-3XHA cut with the same enzymes to yield plasmid pADH1-tc3-3XHA-URA3. This plasmid was used as a template to generate a cassette for integration at a specific promoter using oligonucleotides Tc-CgZCFXX-1 and Tc-CgZCFXX-2. The PCR product was used as a template for a second round of PCR with oligonucleotides Tc-CgZCFXX-3 and Tc-CgZCFXX-4.

### Phenotypic analysis and minimal inhibitory concentration (MIC) assays

Fluconazole and ketoconazole were obtained from Medisca (Montréal, Canada). Micafungin and caspofungin were obtained from Astellas (Markham, Ontario, Canada) and Merck Frost (Kirkland, Québec, Canada), respectively. Sensitivity to drugs was assayed in liquid YPD and on YPD agar plates containing various drugs as detailed in the figures. Strains were grown overnight in liquid YPD medium. The cultures then were diluted at 0.2 OD_600_ and further diluted 5, 25, and 125 times and spotted on appropriate plates. Growth was monitored after 1−2 d. MIC assays were performed as described (Znaidi *et al.* 2007).

## Results and Discussion

**Figure 1:**
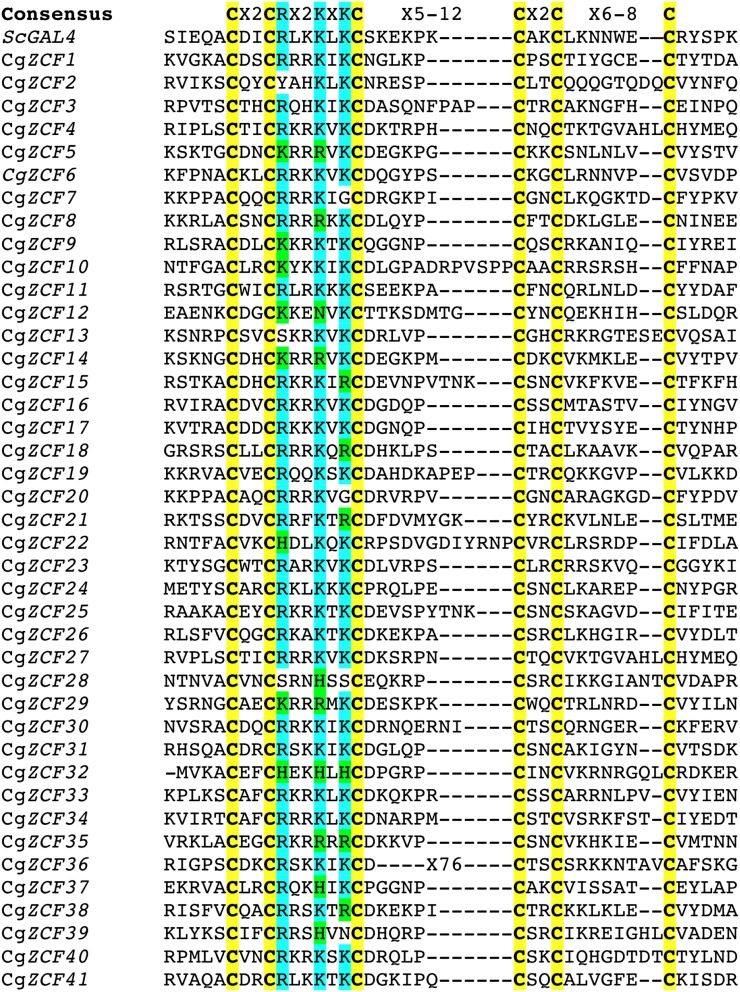
Alignment of the cysteine-rich motif of *S. cerevisiae* Gal4 with *C. glabrata* zinc cluster proteins. *C. glabrata* zinc cluster proteins were identified by BLAST searches of the *C. glabrata* genome using *S. cerevisiae* Gal4 and other zinc cluster proteins as queries and were named Cg*ZCF1* to 41 (*C. glabrata* Zinc Cluster Factor). The cysteines residues (in yellow) of the 41 putative or known zinc cluster proteins were aligned using Gal4 as a reference. A consensus sequence is shown on top of the figure. Some residues (located between the second and third cysteines) are involved in DNA recognition by Gal4 and are shown in turquoise. Conserved or alternate residues found in other *S. cerevisiae* zinc cluster proteins are shown in green. Systematic and gene names are listed in Table 1.

To identify zinc cluster genes in *C. glabrata*, we used Gal4 and related proteins as queries to perform a BLAST search of the *C. glabrata* genome. We identified 41 known or putative zinc cluster genes. Alignments of the various zinc cluster motifs are shown in Figure 1. With the exception of Cg*ZCF36*, the zinc cluster motifs all match the consensus sequence described previously. Cg*ZCF36* has an extended sequence (77 a.a.) between the third and fourth cysteine. However, a similar spacing is found in some *S. cerevisiae* zinc cluster proteins (MacPherson *et al.* 2006), suggesting that Cg*ZCF36* also encodes a zinc cluster protein. In Gal4, the motif Arg-X2.-Lys-X-Lys (where X is any amino acid) is found between the second and third cysteines. The first arginine and the second lysine form salt bridges with phosphate groups in DNA whereas the first lysine is involved in making base-specific contacts (Marmorstein *et al.* 1992). Other *S. cerevisiae* zinc cluster proteins also harbor this motif, even though some of them have, for example, Arg, His, or Asn residues instead of the first Lys. Strikingly, this motif is also found in the vast majority of zinc cluster proteins in *C. glabrata* (Figure 1). In summary, the *C. glabrata* genome contains 41 zinc cluster genes that are highly likely to encode *bona fide* zinc cluster proteins. A list of the *C. glabrata* 41 known or putative zinc cluster genes is provided in Table 1 along with their *S. cerevisiae* homologs.

**Figure 2:**
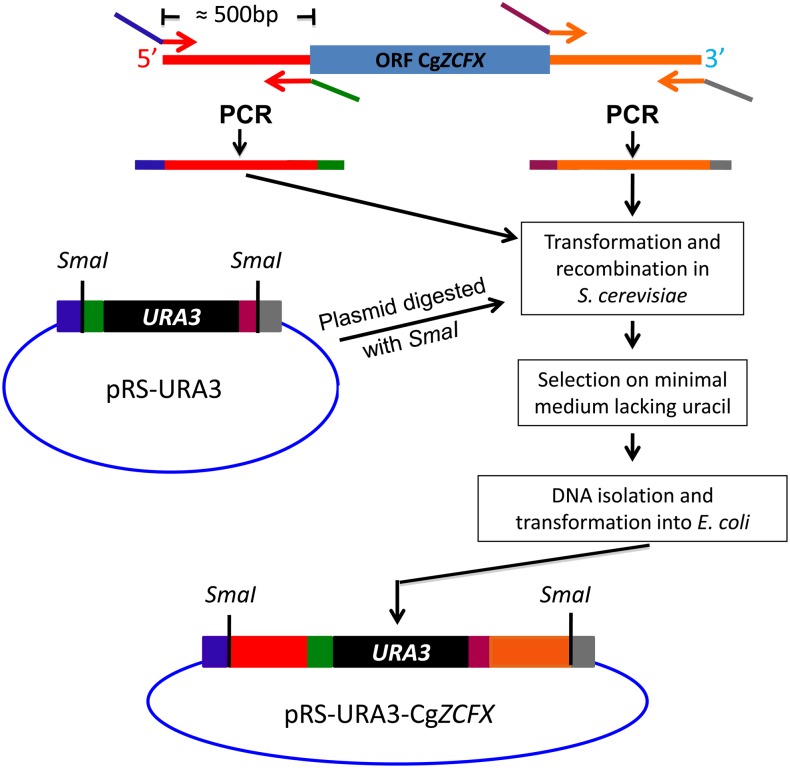
Strategy used to generate cassettes for deletion of zinc cluster genes in *C. glabrata*. Fragments corresponding to sequences flanking an open reading frame (ORF) of interest were amplified by polymerase chain reaction (PCR). Oligos were designed so that they contain 45 bp of homology to the plasmid pRS-URA3 containing the *S. cerevisiae URA3* gene. pRS-URA3 was digested with *SmaI* and transformed along with the two PCR products into *S. cerevisiae*. A quadruple recombination between the plasmid backbone, the PCR products and the *URA3* marker allows the generation of a plasmid which can be recovered and amplified in *E. coli*. After digestion with *Sma*I, the DNA is then transformed into *C. glabrata*.

Strikingly, 36 of 41 zinc cluster genes in *C. glabrata* are uncharacterized (Table 1). To obtain insights into the function of these putative zinc cluster proteins, we generated a panel of deletion strains. To this end, we constructed plasmids containing the *S. cerevisiae URA3* gene flanked by approximately 500 bp of sequences located upstream and downstream of the ORF of a zinc cluster gene of interest (Figure 2). Linearized plasmids were transformed into a Ura^−^ *C*. *glabrata* and transformants were selected on plates lacking uracil. Using this strategy, we successfully deleted 37 of 40 zinc cluster genes (the zinc cluster gene Cg*ZCF41* was not included in the analysis).

**Figure 3:**
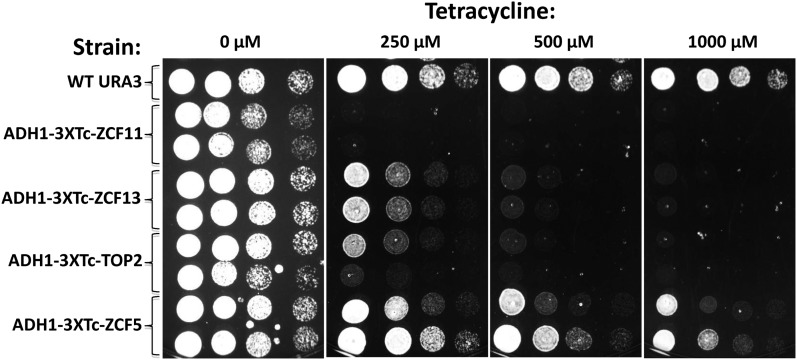
The genes Cg*ZCF11*, Cg*ZCF13*, and Cg*TOP2* are essential. Strains (as listed on the left) were grown overnight in rich medium containing 100 µM tetracycline. Cells were then serially diluted and spotted on plates containing tetracycline at concentrations indicated on the top of the Figure and plates were incubated at 30° for 24 h. Spotting experiments were performed with two independent clones for the genes tested. As a control, a *C. glabrata* ortholog of the essential *S. cerevisiae* gene *TOP2* (encoding topoisomerase II) was used. It is not clear whether Cg*ZCF5* is essential or not because partial growth inhibition could be due, for example, to incomplete translational inhibition.

To test whether the three remaining genes are essential, we adapted a procedure initially developed for *S. cerevisiae* for use in *C. glabrata* (Kotter *et al.* 2009). The natural promoters of the genes of interest were replaced with the *S. cerevisiae* promoter *ADH1* followed by three aptamers (3XTc) that were inserted just upstream of the initiating codon. The RNA aptamers, located in the 5′ UTR, bind with high affinity to tetracycline, resulting in the formation of a secondary structure that prevents translation, thus verifying if a gene is essential (Figure 3). As expected (Kotter *et al.* 2009), the addition of tetracycline did not affect growth of the wild-type strain. As a positive control, we conditionally expressed the topoisomerase CgTop2, a homolog of *S. cerevisiae* Top2 encoded by an essential gene. Inhibition of translation of the Cg*TOP2* mRNA by addition of tetracycline completely abolished growth, thus validating this assay in *C. glabrata*. Similarly, inhibition of *C*gZcf13 (CgCep3) expression prevented growth, in agreement with a study which showed that the Cg*CEP3* gene is essential (Stoyan and Carbon 2004). Our results also show that Cg*ZCF11* is an essential gene, whereas it is not clear whether CgZcf5 (a *S. cerevisiae* ortholog of Upc2/Ecm22) is essential in the strain used for our experiments. The Cg*ZCF5* gene is dispensable in a different strain background (Nagi *et al.* 2011). Thus, only two zinc cluster genes are essential in *C. glabrata*. A similar phenomenon was observed in *S. cerevisiae* where only two zinc cluster genes are essential, including *CEP3* (Akache and Turcotte 2002).

### Phenotypic analysis of strains lacking zinc cluster genes

**Figure 4:**
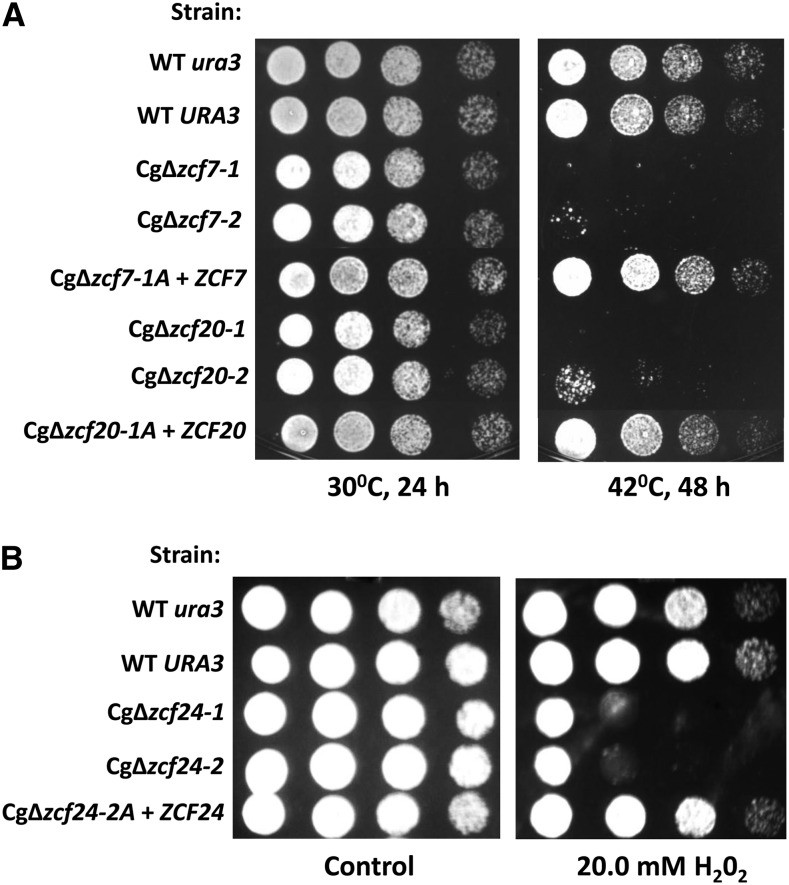
Strains Cg∆*zcf7* and Cg∆*zcf20* are thermosensitive whereas strain Cg∆*zcf24* is sensitive to oxidative stress. Strains were grown overnight in rich medium, serially diluted, and spotted on plates as described in the section *Material and Methods*. (A) Two independent clones of deletion strains Cg∆*zcf7* and Cg∆*zcf20* were tested and are Ura^+^. Cg∆*zcf7-1A* + *ZCF7* and Cg∆*zcf20-1A* + *ZCF20* are deletion strains were a wild-type allele was introduced and the strains are Ura^-^. B) Two independent clones of deletion strain Cg∆*zcf24* were tested and are Ura^+^. Cg∆*zcf24-1A* + *ZCF24* is a deletion strain were a wild-type allele was introduced and the strain is Ura^−^.

Using our panel of deletion strains, we performed phenotypic analysis under various conditions (*e.g.*, high temperature, salt stress, exposure to antifungal drugs, etc.) and phenotypes are listed in Table 2. Phenotypes for a number of deletion strains are described herein whereas data for the remaining strains can be found in Figure S1. In addition, complementation assays using at least two revertant strains for all deleted zinc cluster genes (with the exception of Cg*ZCF9* where only one revertant strain was obtained) confirmed that the observed phenotypes were due to deletion of a given zinc cluster gene and not to secondary mutations (see herein and Figure S1). Two deletion strains, Cg∆*zcf7* and Cg∆*zcf20*, are thermosensitive (Figure 4A). Introduction of wild-type alleles in the deletion strains restored growth at high temperature. One deletion strain (Cg∆*zcf24)* showed high sensitivity to oxidative stress, as assayed with H_2_O_2_ (Figure 4B), in agreement with a previous report (Noble *et al.* 2013). CgZcf24 is highly homologous to *S. cerevisiae* Stb5. We previously showed that deletion of *STB5* results in sensitivity to oxidative stress and that Stb5 is an activator to genes of the pentose phosphate pathway and other genes involved in the production of NADPH, a cofactor involved in conferring resistance to oxidative stress (Larochelle *et al.* 2006). CgStb5 does not appear, however, to regulate genes of the pentose phosphate pathway (data not shown), in agreement Noble *et al.* (2013). It will be interesting to determine the reason for the sensitivity to oxidative stress of cells lacking Cg*ZCF24*.

**Table 2:** Summary of the phenotypes observed for strains carrying deletions of zinc cluster genes

Zinc Cluster Gene Deleted	Fluconazole	Ketoconazole	Micafungin	H_2_O_2_	42°	Caffeine	LiCl	SDS
Cg*ZCF1* (*PDR1*)	Highly sens.	Highly sens.	Not tested	Not tested	Not tested	Not tested	Not tested	Not tested
Cg*ZCF4*	Slightly sens.	Sens.					Res.	
Cg*ZCF6*			Res.					Sens.
Cg*ZCF7*				Slightly sens.	Sens.	Sens.	Sens.	
Cg*ZCF9*	Slightly sens.	Sens.						
Cg*ZCF10*	Slightly sens.	Sens.					Res.	
Cg*ZCF12*	Slightly sens.	Sens.						
Cg*ZCF1*6	Slightly sens.	Sens						
Cg*ZCF17*							Res.	
Cg*ZCF18*	Slightly sens.	Sens.						
Cg*ZCF20*				Slightly sens.	Sens.	Sens.	Slightly sens.	
Cg*ZCF23*	Slightly sens.	Sens.						
Cg*ZCF24* (*STB5*)	Slightly sens.	Sens.	Slightly sens.	Sens.			Res.	
Cg*ZCF26*	Slightly sens.	Slightly sens.					Res.	
Cg*ZCF27*	Slightly sens..	Sens.						
Cg*ZCF29*	Slightly sens.	Sens.						
Cg*ZCF31*	Slightly sens.	Sens.						
Cg*ZCF33*	Slightly sens.	Sens.						
Cg*ZCF36*	Slightly sens.	Sens.					Res.	
Cg*ZCF37*	Slightly sens.	Sens.					Res.	
Cg*ZCF39*	Slightly sens.	Slightly sens.					Res.	

For azoles compounds, a deletion strain was scored as sensitive if the MIC difference with the wild-type stain was 2 or more (see Table 3). *CgZCF5*, Cg*ZCF41*, and the essential genes Cg*ZCF11*, Cg*ZCF13* were not included in the phenotypic analysis. See the *Results* section as well as Figure S1 for spotting assays. SDS, sodium dodecyl sulfate; Sens., sensitive; Res., resistant.

**Figure 5:**
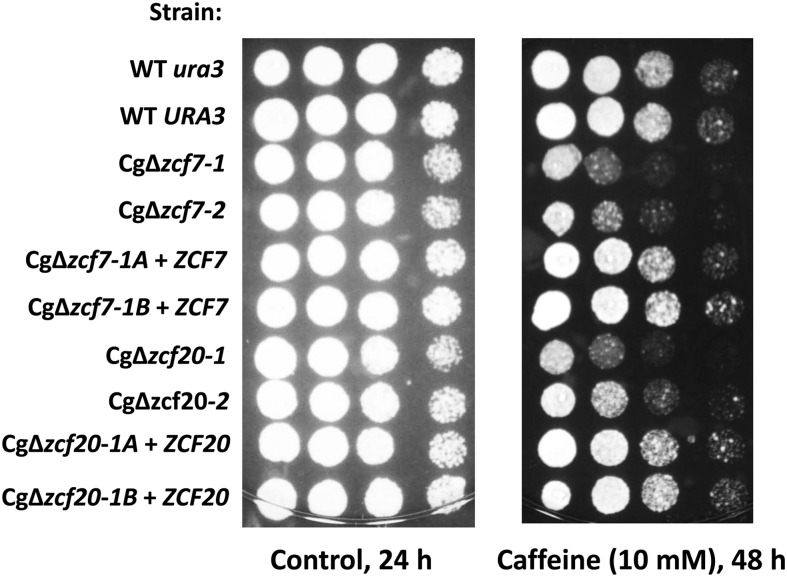
Sensitivity of deletion strains to caffeine. Strains were grown overnight in rich medium, serially diluted and spotted on plates as described in the section *Material and Methods*. Two independent clones of deletion strains Cg∆*zcf7* and Cg∆*zcf20* were tested and are Ura^+^. Cg∆*zcf7-1A* + *ZCF7*, Cg∆*zcf7-1B* + *ZCF7*, Cg∆*zcf20-1A* + *ZCF20*, and Cg∆*zcf20-1B* + *ZCF20* are deletion strains were a wild-type allele was introduced and the strains are Ura^−^.

**Figure 6:**
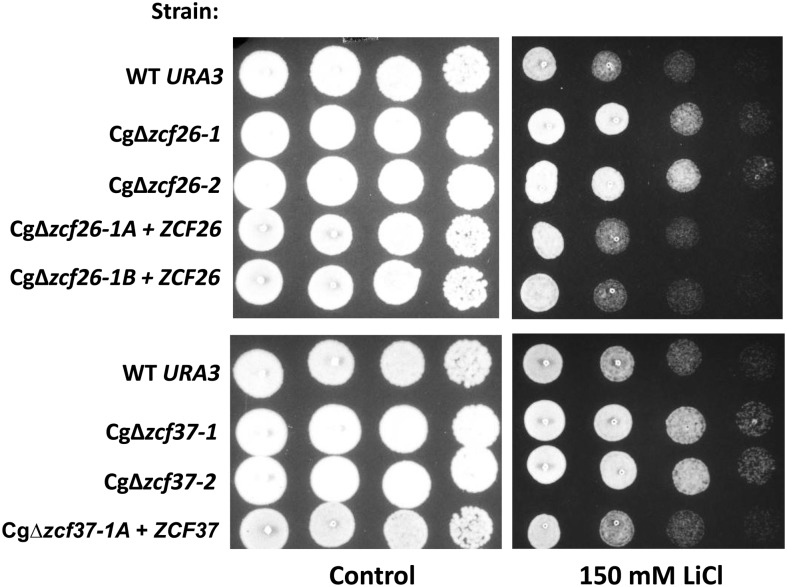
Strains Cg∆*zcf26* and Cg∆*zcf37* show increased tolerance to salt stress. Strains were grown overnight in rich medium, serially diluted and spotted on plates as described in the section *Material and Methods*. Two independent clones of deletion strains Cg∆*zcf26* and Cg∆*zcf37* were tested and are Ura^+^. Cg∆*zcf26-1A* + *ZCF26*, Cg∆*zcf26-1B* + *ZCF26*, and Cg∆*zcf37-1A* + *ZCF37* are deletion strains were a wild-type allele was introduced and the strains are Ura^−^.

We also tested deletion strains for sensitivity to caffeine, an inhibitor of the target of rapamycin pathway (Reinke *et al.* 2006). Cells lacking Cg*ZCF7* or Cg*ZCF20* were sensitive to caffeine (Figure 5) whereas reintroduction of the wild-type alleles in the deletion strains resulted in a wild-type phenotype. High concentrations of sorbitol cause osmotic stress and activation of the high-osmolarity glycerol pathway (Saito and Posas 2012). However, sensitivity to sorbitol was not observed in our screen. Regarding tolerance to salt (150 mM LiCl), only one deletion strain (Cg∆*zcf7*) showed sensitivity under this condition (Figure S1). Unexpectedly, our results show that deletion of eight zinc cluster genes (Cg*ZCF4*, Cg*ZCF10*, Cg*ZCF17*, Cg*ZCF24*, Cg*ZCF26*, Cg*ZCF36*, Cg*ZCF37*, and Cg*ZCF39*) rather results in increased tolerance to salt stress (Figure 6 and Figure S1).

**Figure 7:**
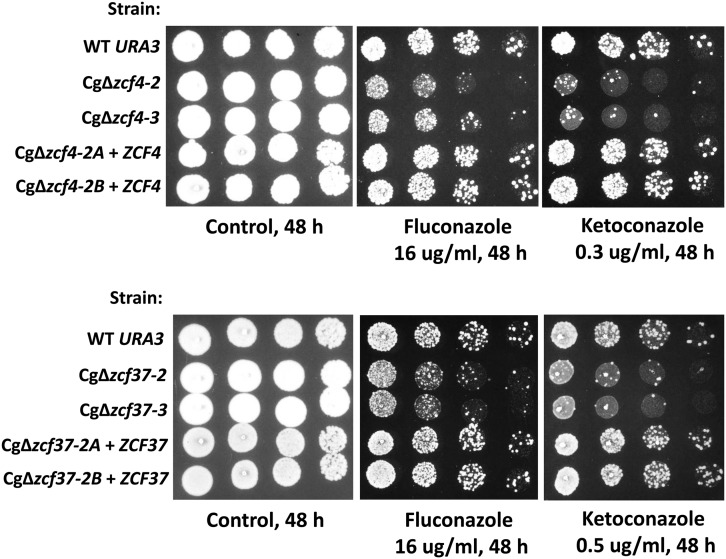
Increased susceptibility of deletion strains Cg∆*zcf4* and Cg∆*zcf37* to azoles. Strains were grown overnight in rich medium, serially diluted, and spotted on plates with or without drugs as indicated in the figure. Two independent clones of deletion strains Cg∆*zcf4* and Cg∆*zcf37* were tested and are Ura^+^. Cg∆*zcf4-2A* + *ZCF4*, Cg∆*zcf4-2B* + *ZCF4*, Cg∆*zcf37-2A* + *ZCF37* and Cg∆*zcf37-2B* + *ZCF37* are deletion strains were a wild-type allele was introduced and the strains are Ura^−^.

Phenotypic analysis also was performed with antifungal drugs. Azoles, such as fluconazole or ketoconazole, are fungistatic antifungal drugs. These compounds target lanosterol 14α-demethylase involved in the synthesis of ergosterol and this enzyme is encoded by the *ERG11* gene. Antifungal activity is caused by decreased ergosterol levels and increased production of toxic ergosterol derivatives (Lupetti *et al.* 2002). As expected (Vermitsky *et al.* 2006; Vermitsky and Edlind 2004), deletion of Cg*PDR1* (Cg*ZCF1*) greatly increased susceptibility to fluconazole and ketoconazole (≥eightfold difference in MIC, Table 3). Unexpectedly, increased susceptibility to azoles (in particular ketoconazole) was observed in many deletion strains (total of 15, Table 3). These strains showed a twofold reduced MIC compared with the wild-type strain. Figure 7 shows spotting assays for deletion strains Cg∆*zcf4* and Cg*∆zcf37*. In agreement with MIC values, both strains showed increased susceptibility to ketoconazole, whereas slightly increased susceptibility was observed for fluconazole. We also note the presence of some small colonies in the presence of fluconazole or ketoconazole. These colonies are probably resistant to the azoles due, for instance, to mutations in Cg*PDR1* (Tsai *et al.* 2006).

**Table 3:** MIC values for fluconazole and ketoconazole as measured in various deletion strains

Strain	MIC Fluconazole, µg/mL	Fold Difference	MIC Ketoconazole, µg/mL	Fold Difference
WT *URA3*	32	N/A	0.5	N/A
WT *ura3*	32	N/A	0.5	N/A
Cg∆*zcf1 (Cg∆pdr1*)	4	8	No growth with 0.03 µg/mL	>8
Cg∆*zcf*4	32	N/A	0.25	2
Cg∆*zcf9*	Not tested	N/A	0.25	2
Cg∆*zcf10*	Not tested	N/A	0.25	2
Cg∆*zcf12*	Not tested	N/A	0.25	2
Cg∆*zcf16*	Not tested	N/A	0.25	2
Cg∆*zcf18*	Not tested	N/A	0.25	2
Cg∆*zcf23*	32	N/A	0.25	2
Cg∆*zcf24*	32	N/A	0.25	2
Cg∆*zcf26*	32	N/A	0.5	N/A
Cg∆*zcf27*	16-26	≈ 2	0.25	2
Cg∆*zcf29*	32	N/A	0.25	2
Cg∆*zcf31*	32	N/A	0.25	2
Cg∆*zcf33*	32	N/A	0.25	2
Cg∆*zcf36*	Not tested	N/A	0.25	2
Cg∆*zcf37*	Not tested	N/A	0.25	2
Cg∆*zcf39*	32	N/A	0.5	N/A

Deletion strains that showed sensitivity to azoles with spotting assays were used to perform MIC assays. MIC, minimal inhibitory concentration; WT, wild type; N/A, not applicable.

**Figure 8:**
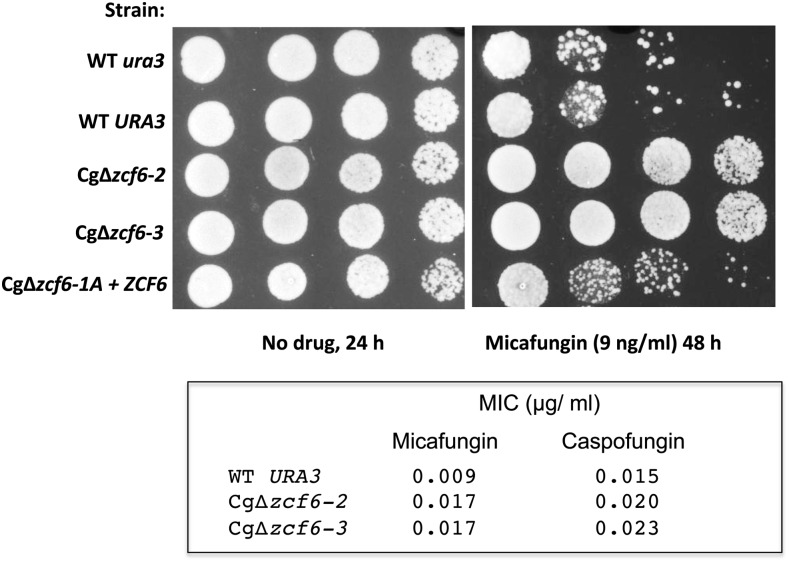
Strain Cg∆*zcf6* shows reduced susceptibility to micafungin. Strains were grown overnight in rich medium, serially diluted, and spotted on plates as described in the section *Material and Methods*. Two independent clones of deletion strains Cg∆*zcf6* were tested and are Ura^+^. Cg∆*zcf6-1A* + *ZCF6* is a deletion strain were a wild-type allele was introduced and the strain is Ura^-^. MIC values are given at the bottom of the figure.

Echinocandins (*e.g.*, caspofungin, micafungin) are the latest class of antifungal drugs used in the clinic, and they have fungicidal activity (reviewed in Chen *et al.* 2011 and Mayr *et al.* 2011). Echinocandins inhibit the activity of a two-subunit enzyme involved in the synthesis of the polysaccharide 1,3-β-glucan, which is a major and essential component of the cell wall. In *S. cerevisiae*, one subunit is encoded by the genes *FKS1*, *FKS2*, and *FKS3* whereas the second one is encoded by *RHO1*. Resistance to echinocandins has been attributed to mutations in the *FKS1* and *FKS2* genes (Johnson *et al.* 2011; Kahn *et al.* 2007; Katiyar *et al.* 2006; Thompson *et al.* 2008). A strain carrying a deletion of Cg*ZCF24* showed slightly increased susceptibility to micafungin (data not shown). Interestingly, deletion of Cg*ZCF6* resulted in reduced susceptibility to micafungin, as determined by spotting assays (Figure 8, top panel). Moreover, with a Cg∆*zcf6* strain, we observed a twofold increase in MIC for micafungin whereas only a slight difference was observed with caspofungin (Figure 8, bottom panel). The Cg∆*zcf6* strain is also sensitive to 0.04% sodium dodecyl sulfate (data not shown), a phenotype that is indicative of cell wall defects. These phenotypes were also observed using a Cg∆*zcf6* deletion strain generated with the dominant marker SAT1 instead of *URA3* (Figure S1).

The *S. cerevisiae* zinc cluster protein Ppr1 is highly homologous to CgZcf6 (*P*-value 1.6 X10^−229^). Ppr1 is an activator of the *URA* genes involved in pyrimidine synthesis (Losson and Lacroute 1981; MacPherson *et al.* 2006). However, it is not clear whether the two factors perform the same function. For example, a ∆*ppr1* strain does not show altered susceptibility to micafungin (data not shown). Transcription factor rewiring may explain the apparent functional difference between CgZcf6 and ScPpr1. It will be interesting to determine the molecular basis for the decreased susceptibility to micafungin of a Cg∆*zcf6* strain.

In this study, we have performed phenotypic analysis of a *C. glabrata* family of transcriptional regulators, the zinc cluster proteins. Results show that only two zinc cluster genes are essential (Figure 3). Their gene products may be potential targets for antifungal drugs because zinc cluster proteins are fungal (and amoebae) specific. Phenotypes have been identified for more than half of the zinc cluster genes, strongly suggesting that these genes do encode functional proteins. However, we were unable to assign phenotypes for a number of zinc cluster proteins. Some of them may perform functions related to a specific environment (*e.g.*, survival in macrophages) or may show redundancy. In summary, our panel of deletion strains along with our phenotypic analysis will provide useful tools to the researcher community for the study of this family of regulators in an important fungal pathogen.

## 

## Supplementary Material

Supporting Information
