# Supplementary material for: Phenotypic Analysis of a Family of Transcriptional Regulators, the Zinc Cluster Proteins, in the Human Fungal Pathogen Candida glabrata
Source: G3 (Bethesda). 2014 Mar 21;4(5):931–40. doi: 10.1534/g3.113.010199 (PMC4025492; doi:10.1534/g3.113.010199)
Supplement: Supporting Information [file supp_4_5_931__index.html]

Phenotypic Analysis of a Family of Transcriptional Regulators, the Zinc Cluster Proteins, in the Human Fungal Pathogen Candida glabrata — Supporting Information 

# Phenotypic Analysis of a Family of Transcriptional Regulators, the Zinc Cluster Proteins, in the Human Fungal Pathogen *Candida glabrata*

## Supporting Information for Klimova *et al.*, 2014

**Files in this Data Supplement:**

- Supporting Information - Figure S1 and Tables S1-S2 (PDF, 3 MB)
- Figure S1 - Additional phenotypes for strains carrying deletions of zinc cluster genes. (PDF, 3 MB)
- Table S1 - List of oligonucleotides used in this study (.xls, 63 KB)
- Table S2 - List of *C. glabrata* strains used in this study. All strains are derived from strain 66032*ura3* (Vermitsky *et al.* 2006). (.xlsx, 18 KB)
