# Supplementary material for: Phenotypic Analysis of a Family of Transcriptional Regulators, the Zinc Cluster Proteins, in the Human Fungal Pathogen Candida glabrata
Source: G3 (Bethesda). 2014 Mar 21;4(5):931–40. doi: 10.1534/g3.113.010199 (PMC4025492; doi:10.1534/g3.113.010199)
Supplement: Supporting Information [file supp_g3.113.010199_010199SI.pdf]

G3·Genes | Genomes | Genetics

Phenotypic analysis of a family of transcriptional regulators, the zinc cluster proteins, in the human fungal pathogen *Candida glabrata*

Natalia Klimova[*], Ralph Yeung[§], Nadezda Kachurina[*], and Bernard Turcotte[* § #]

Depts. of Medicine[*], Biochemistry[§], and Microbiology and Immunology[#]
McGill University Health Centre
McGill University
Montréal, Québec
Canada H3A 1A1

Corresponding author
Bernard Turcotte
Room H5.74
Royal Victoria Hospital
687 Pine Ave. West
Montréal, Québec
Canada
H3A 1A1

DOI: 10.1534/g3.113.010199

Tolerance to salt (150 mM LiCl)

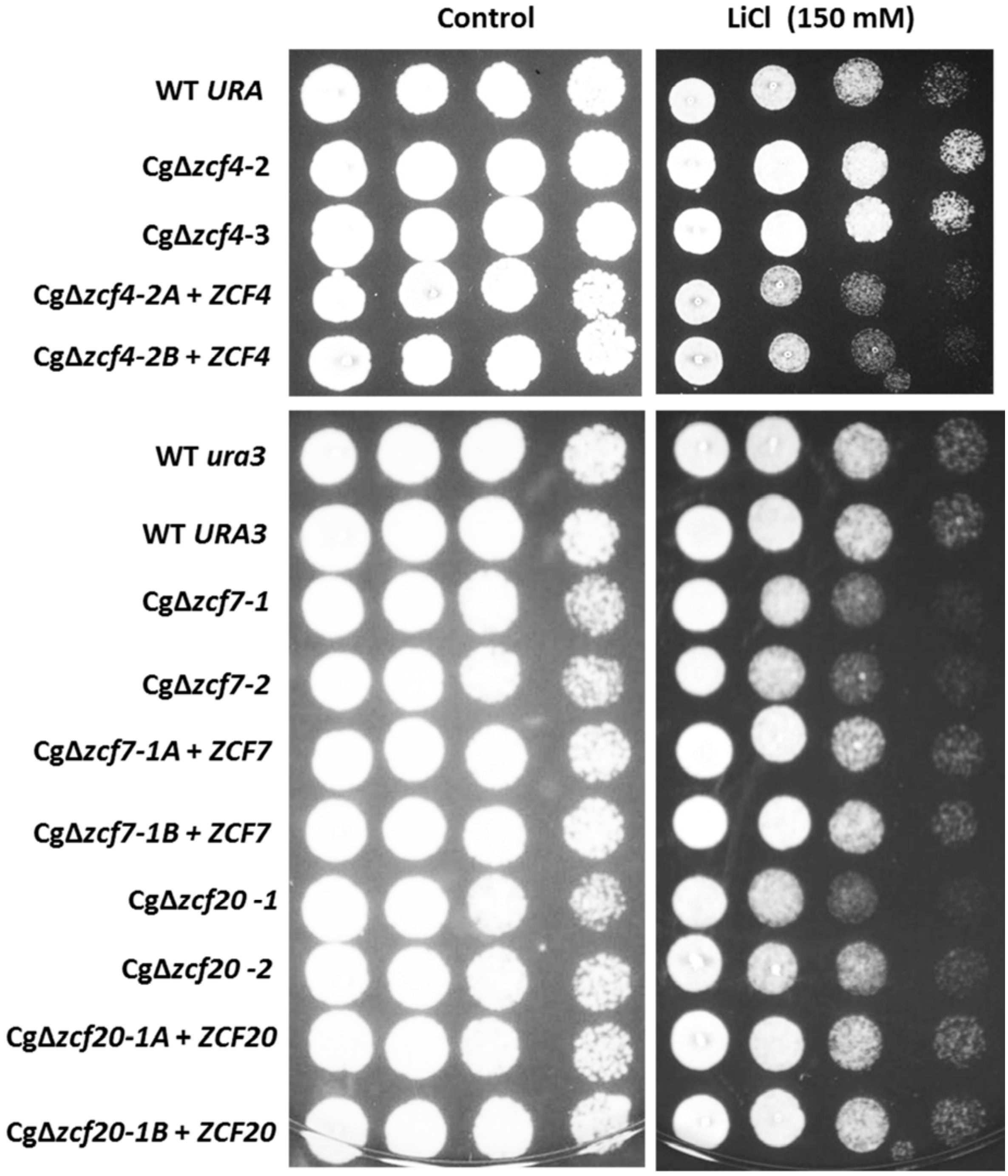

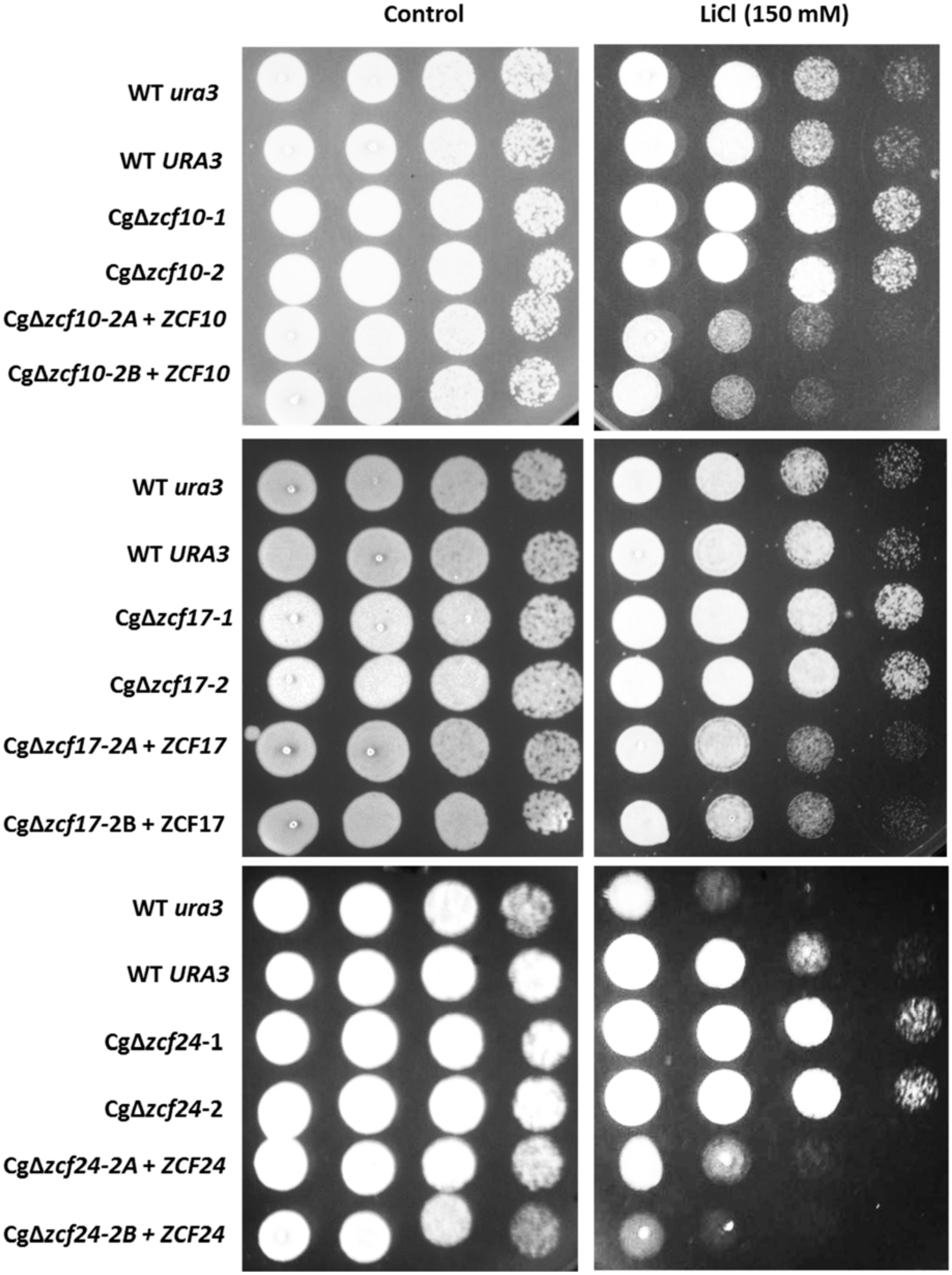
Control
LiCl (150 mM)
WT ura3
WT URA3
CgΔzcf10-1
CgΔzcf10-2
CgΔzcf10-2A + ZCF10
CgΔzcf10-2B + ZCF10
WT ura3
WT URA3
CgΔzcf17-1
CgΔzcf17-2
CgΔzcf17-2A + ZCF17
CgΔzcf17-2B + ZCF17
WT ura3
WT URA3
CgΔzcf24-1
CgΔzcf24-2
CgΔzcf24-2A + ZCF24
CgΔzcf24-2B + ZCF24

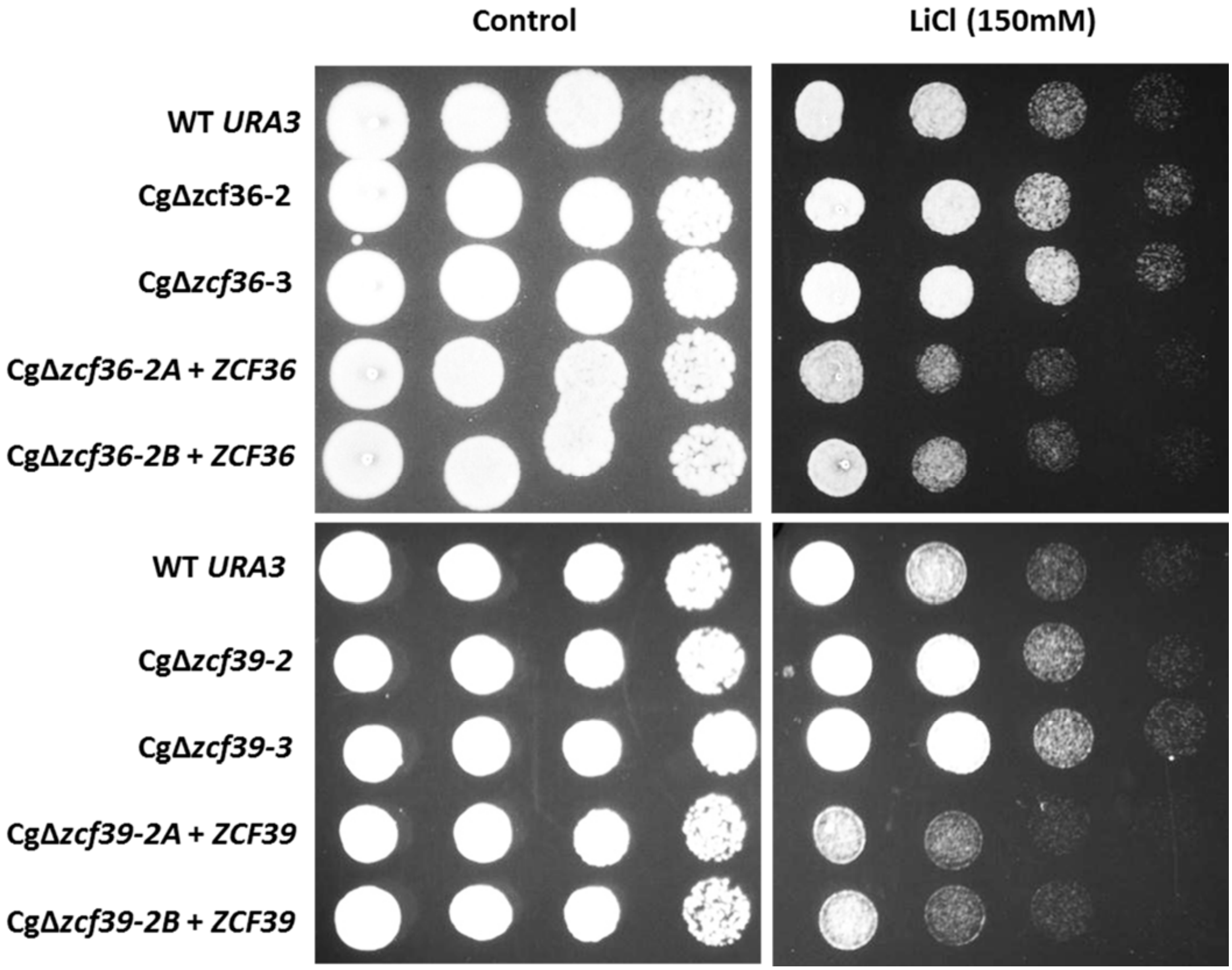
Control
LiCl (150mM)
WT URA3
CgΔzcf36-2
CgΔzcf36-3
CgΔzcf36-2A + ZCF36
CgΔzcf36-2B + ZCF36
WT URA3
CgΔzcf39-2
CgΔzcf39-3
CgΔzcf39-2A + ZCF39
CgΔzcf39-2B + ZCF39

Sensitivity to ketoconazole

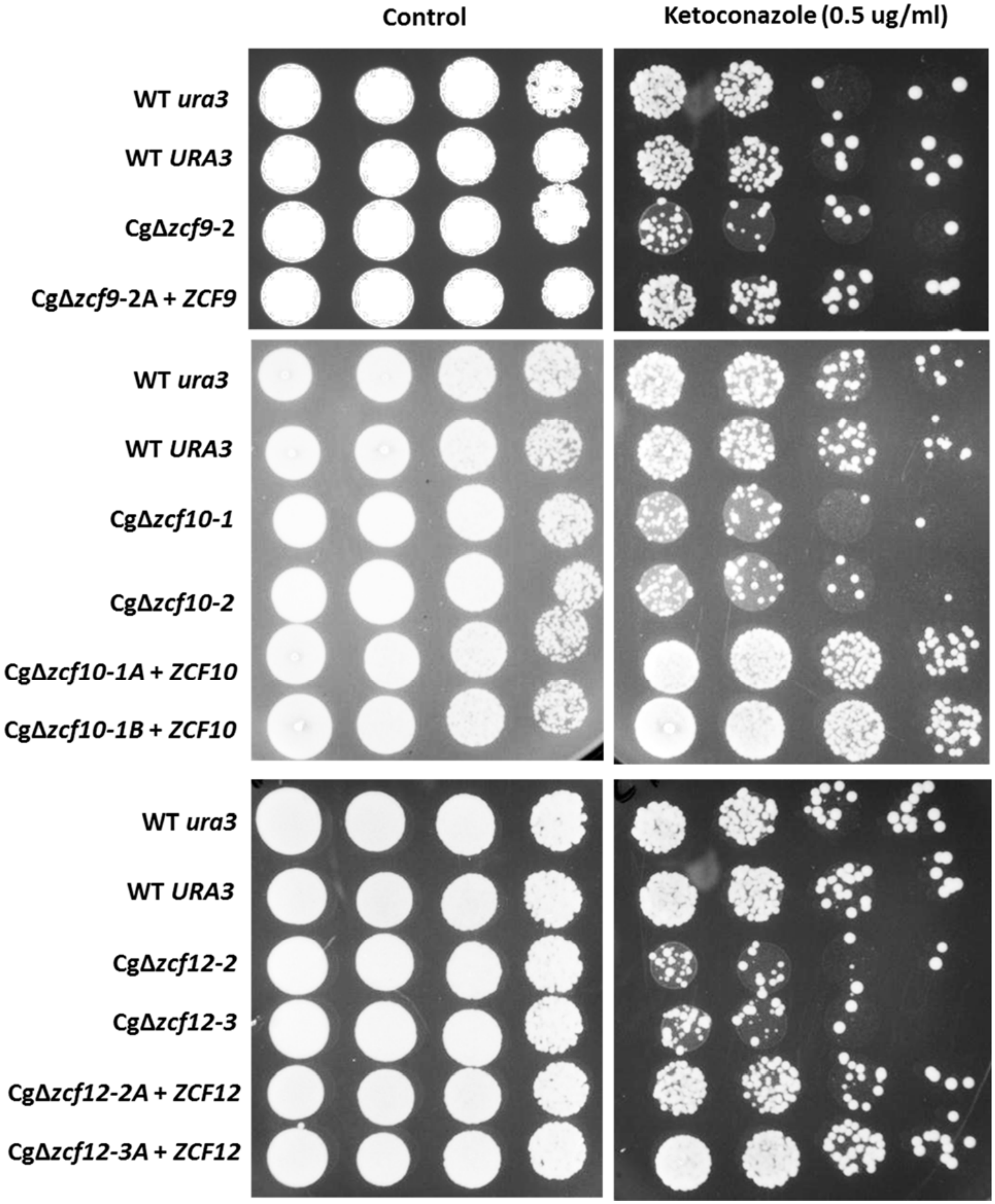

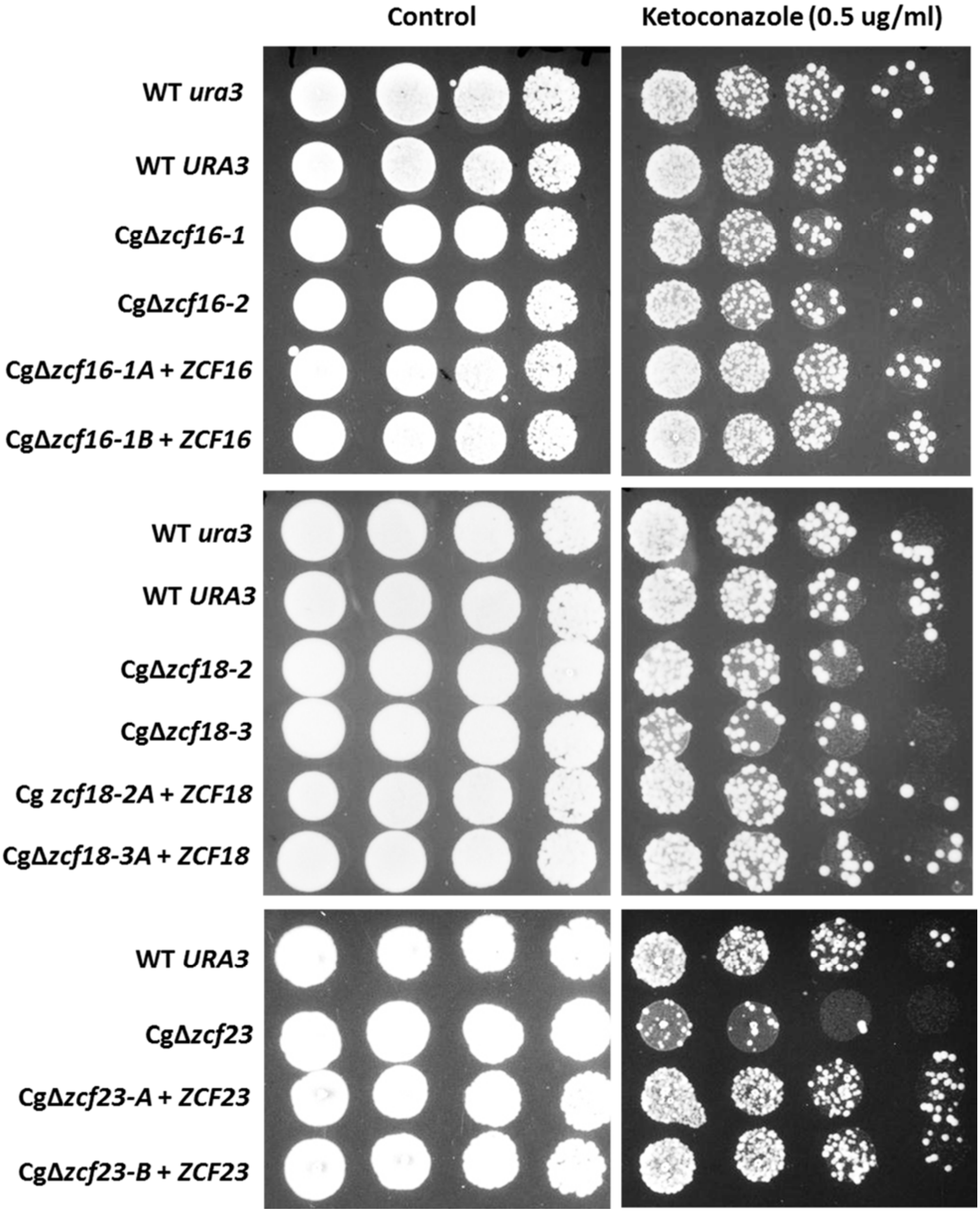
Control
Ketoconazole (0.5 ug/ml)
WT *ura3*
WT *URA3*
CgΔ*zcf16-1*
CgΔ*zcf16-2*
CgΔ*zcf16-1A* + *ZCF16*
CgΔ*zcf16-1B* + *ZCF16*
WT *ura3*
WT *URA3*
CgΔ*zcf18-2*
CgΔ*zcf18-3*
Cg *zcf18-2A* + *ZCF18*
CgΔ*zcf18-3A* + *ZCF18*
WT *URA3*
CgΔ*zcf23*
CgΔ*zcf23-A* + *ZCF23*
CgΔ*zcf23-B* + *ZCF23*

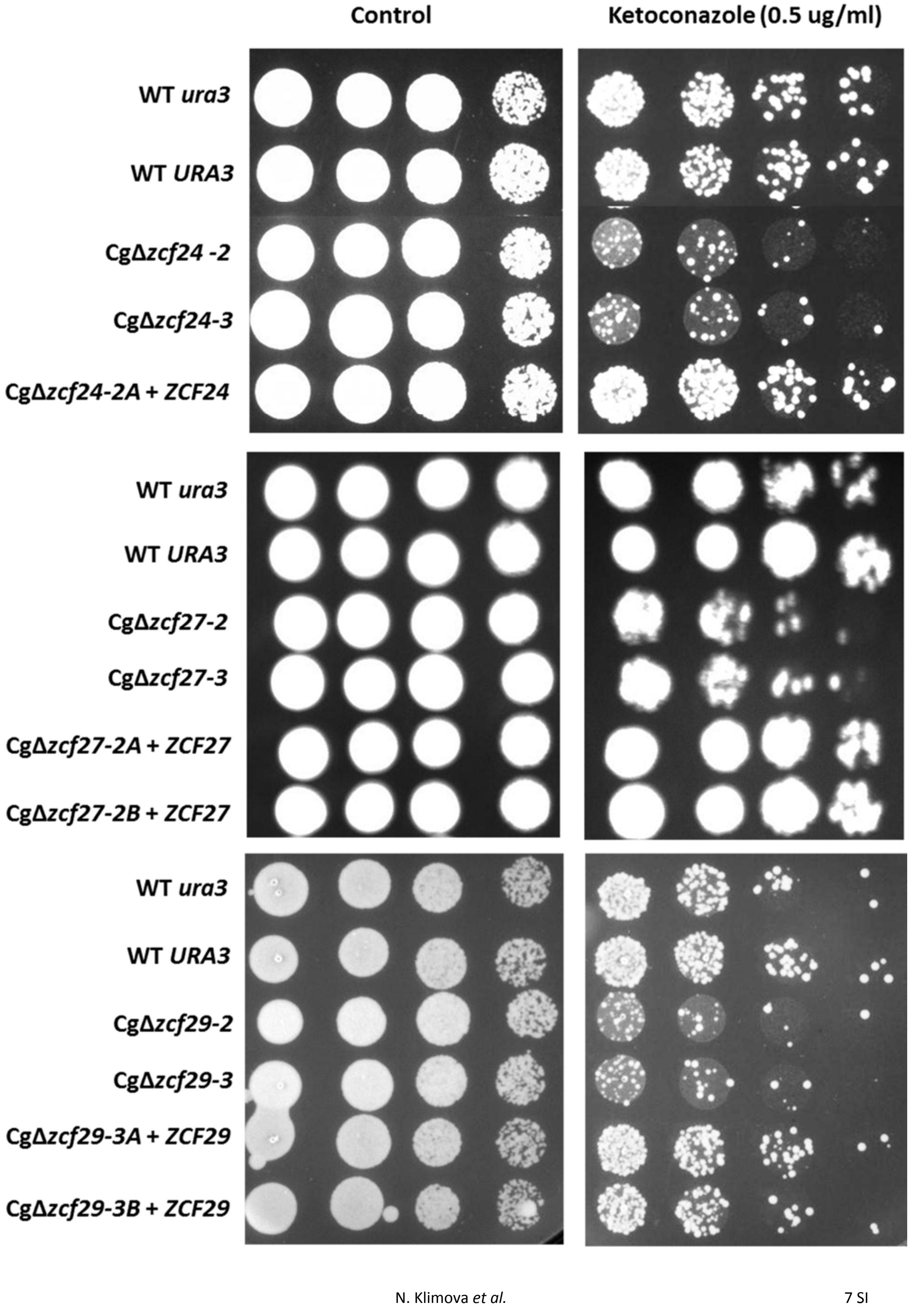
Control
Ketoconazole (0.5 ug/ml)
WT ura3
WT URA3
CgΔzcf24 -2
CgΔzcf24-3
CgΔzcf24-2A + ZCF24
WT ura3
WT URA3
CgΔzcf27-2
CgΔzcf27-3
CgΔzcf27-2A + ZCF27
CgΔzcf27-2B + ZCF27
WT ura3
WT URA3
CgΔzcf29-2
CgΔzcf29-3
CgΔzcf29-3A + ZCF29
CgΔzcf29-3B + ZCF29

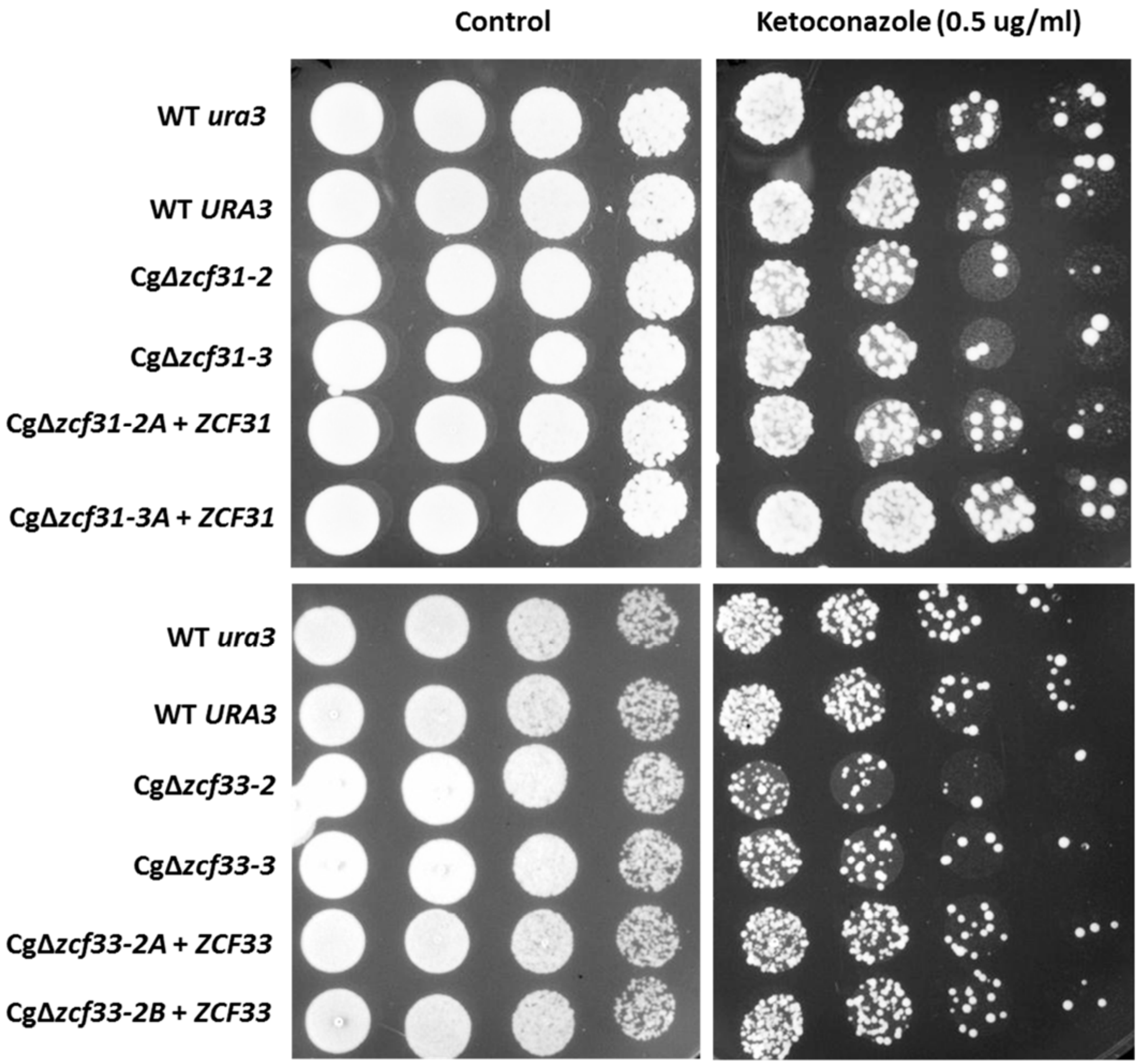
Control
Ketoconazole (0.5 ug/ml)
WT ura3
WT URA3
CgΔzcf31-2
CgΔzcf31-3
CgΔzcf31-2A + ZCF31
CgΔzcf31-3A + ZCF31
WT ura3
WT URA3
CgΔzcf33-2
CgΔzcf33-3
CgΔzcf33-2A + ZCF33
CgΔzcf33-2B + ZCF33

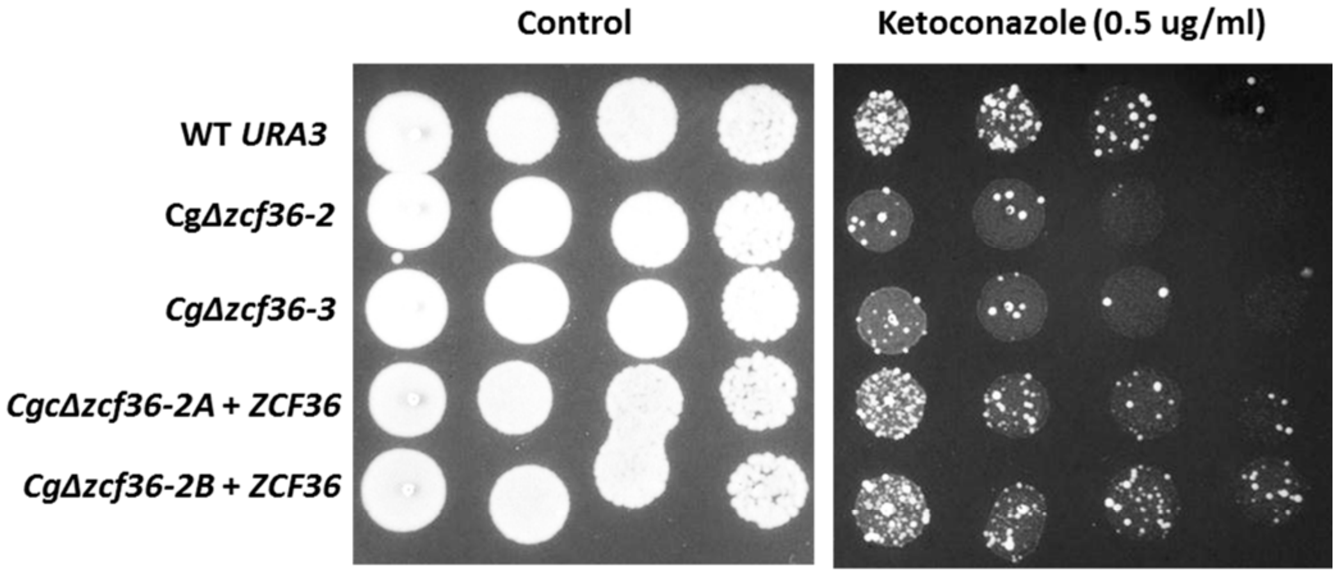

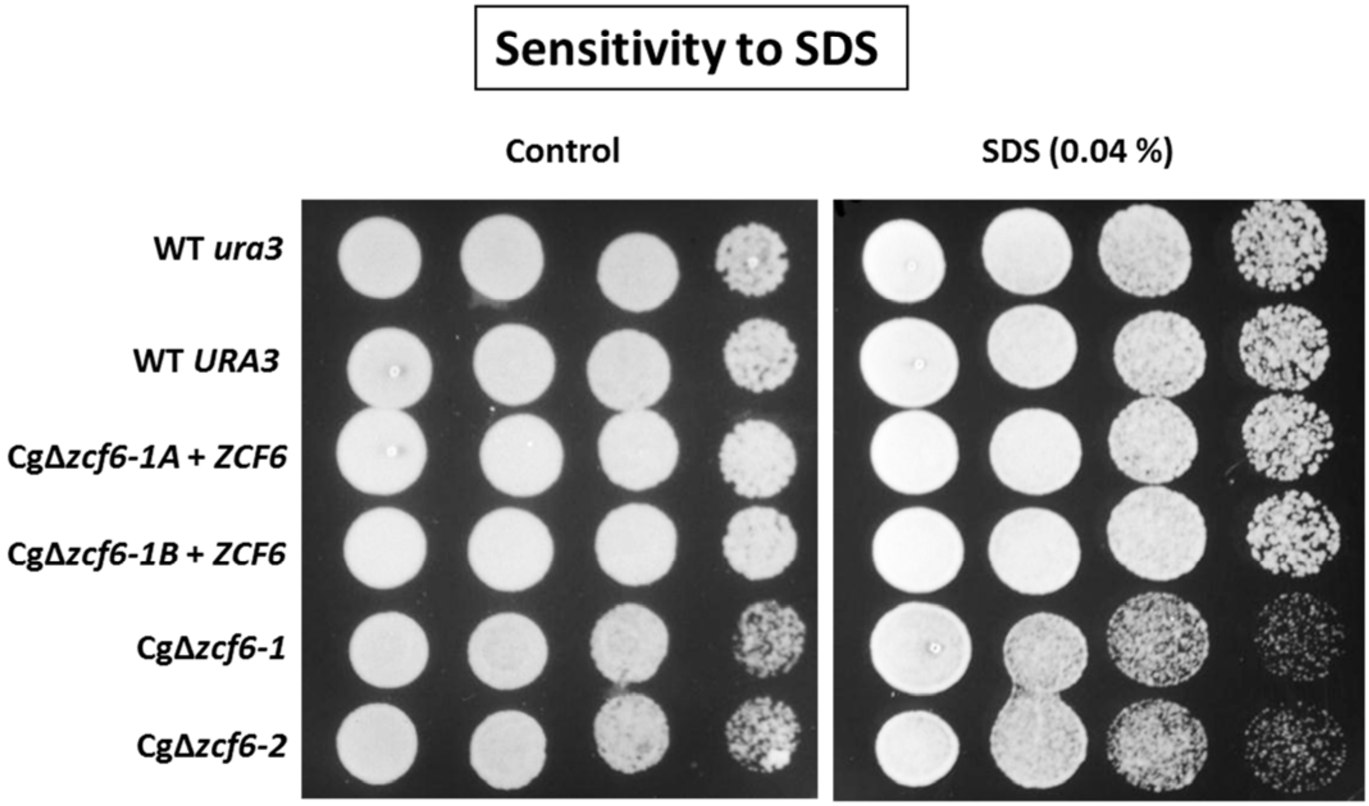

Figure S1 Additional phenotypes for strains carrying deletions of zinc cluster genes. Strains were grown overnight in rich medium, serially diluted and spotted on plates as described in Material and Methods. All deletion strains a*re* Ura^{+}. "CgΔ*zcfxx* + *ZCFXX*" are deletion strains where a wild-type allele for the zinc cluster gene *XX* has been reintroduced for complementation assays. CgΔ*zcfxx* + *ZCFXX* strains are Ura^{-}. Page 2 SI: Altered tolerance to salt (150mM LiCl). Page 5 SI: Susceptibility to ketoconazole. Page 9 SI: Sensitivity to SDS (0.04%).

Tables S1-S2
Available for download as Excel files at http://www.g3journal.org/lookup/suppl/doi:10.1534/g3.113.010199/-/DC1

Table S1 List of oligonucleotides used in this study

Table S2 List of *C. glabrata* strains used in this study. All strains are derived from strain 66032*ura3* (VERMITSKY *et al.* 2006).
