# Supplementary material for: Phenotypic Analysis of a Family of Transcriptional Regulators, the Zinc Cluster Proteins, in the Human Fungal Pathogen Candida glabrata
Source: G3 (Bethesda). 2014 Mar 21;4(5):931–40. doi: 10.1534/g3.113.010199 (PMC4025492; doi:10.1534/g3.113.010199)
Supplement: Supporting Information [file supp_g3.113.010199_FigureS1.pdf]

Tolerance to salt (150 mM LiCl)

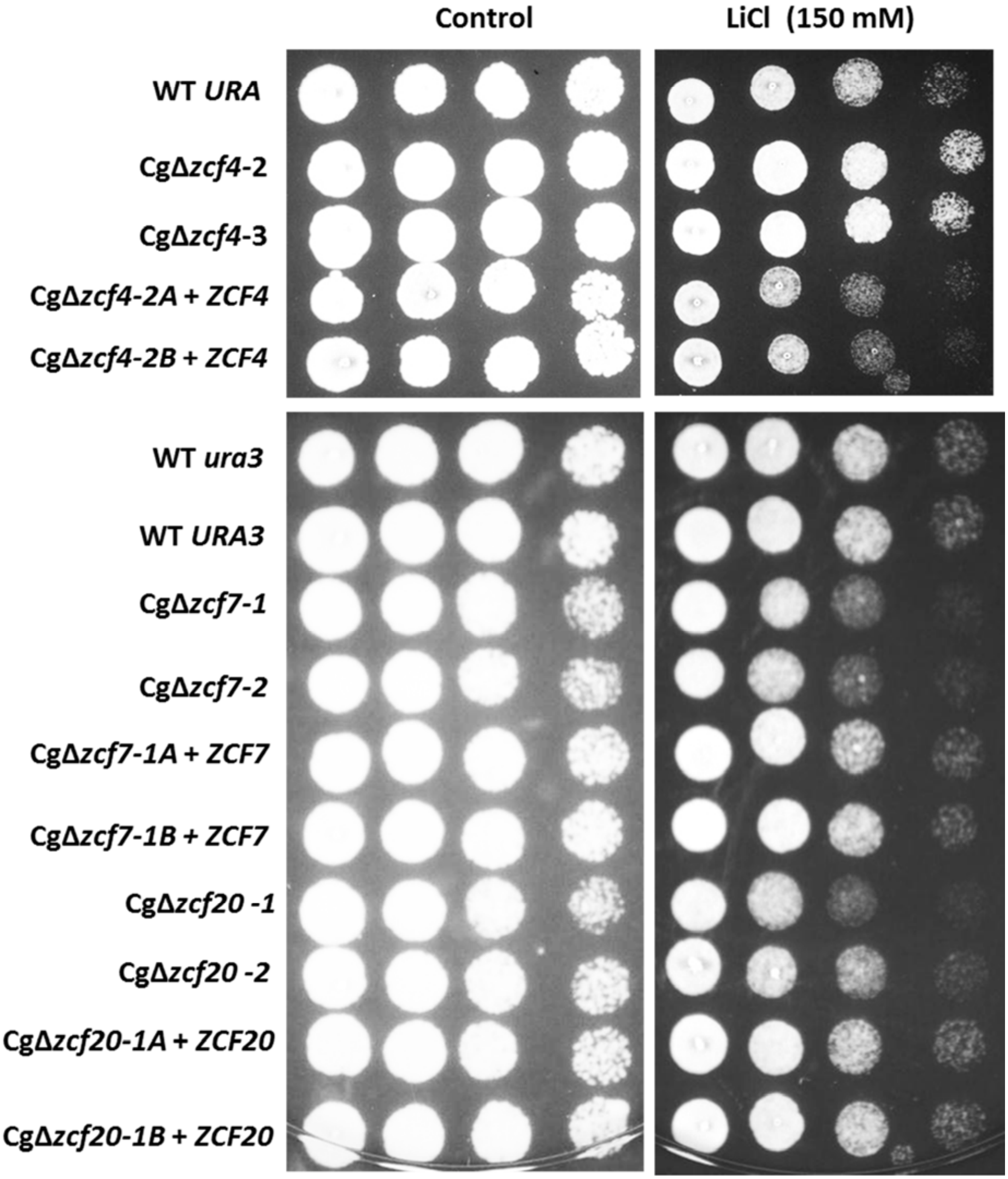

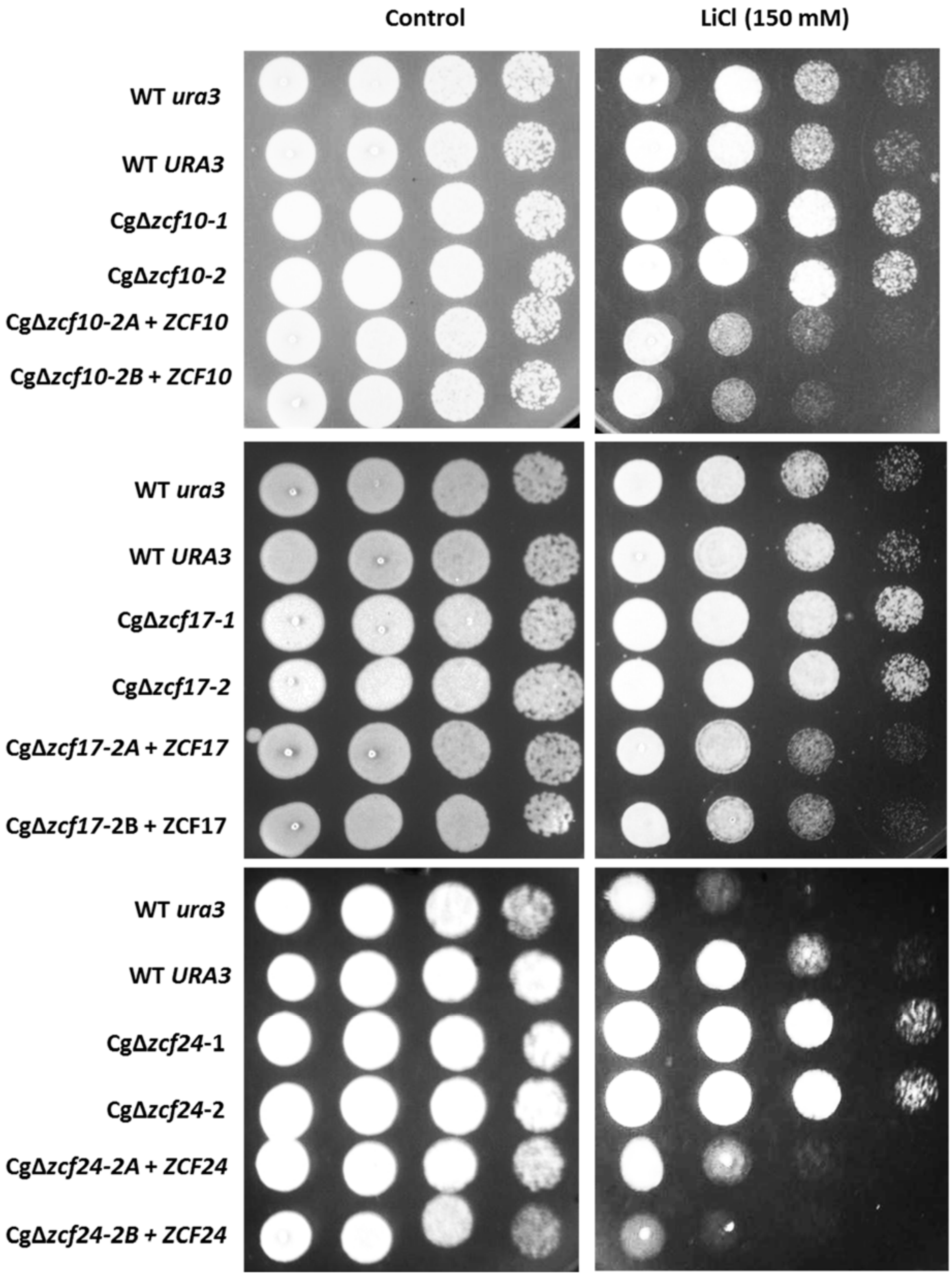
Control
LiCl (150 mM)
WT *ura3*
WT *URA3*
CgΔ*zcf10-1*
CgΔ*zcf10-2*
CgΔ*zcf10-2A + ZCF10*
CgΔ*zcf10-2B + ZCF10*
WT *ura3*
WT *URA3*
CgΔ*zcf17-1*
CgΔ*zcf17-2*
CgΔ*zcf17-2A + ZCF17*
CgΔ*zcf17*-2B + ZCF17
WT *ura3*
WT *URA3*
CgΔ*zcf24*-1
CgΔ*zcf24*-2
CgΔ*zcf24-2A + ZCF24*
CgΔ*zcf24-2B + ZCF24*

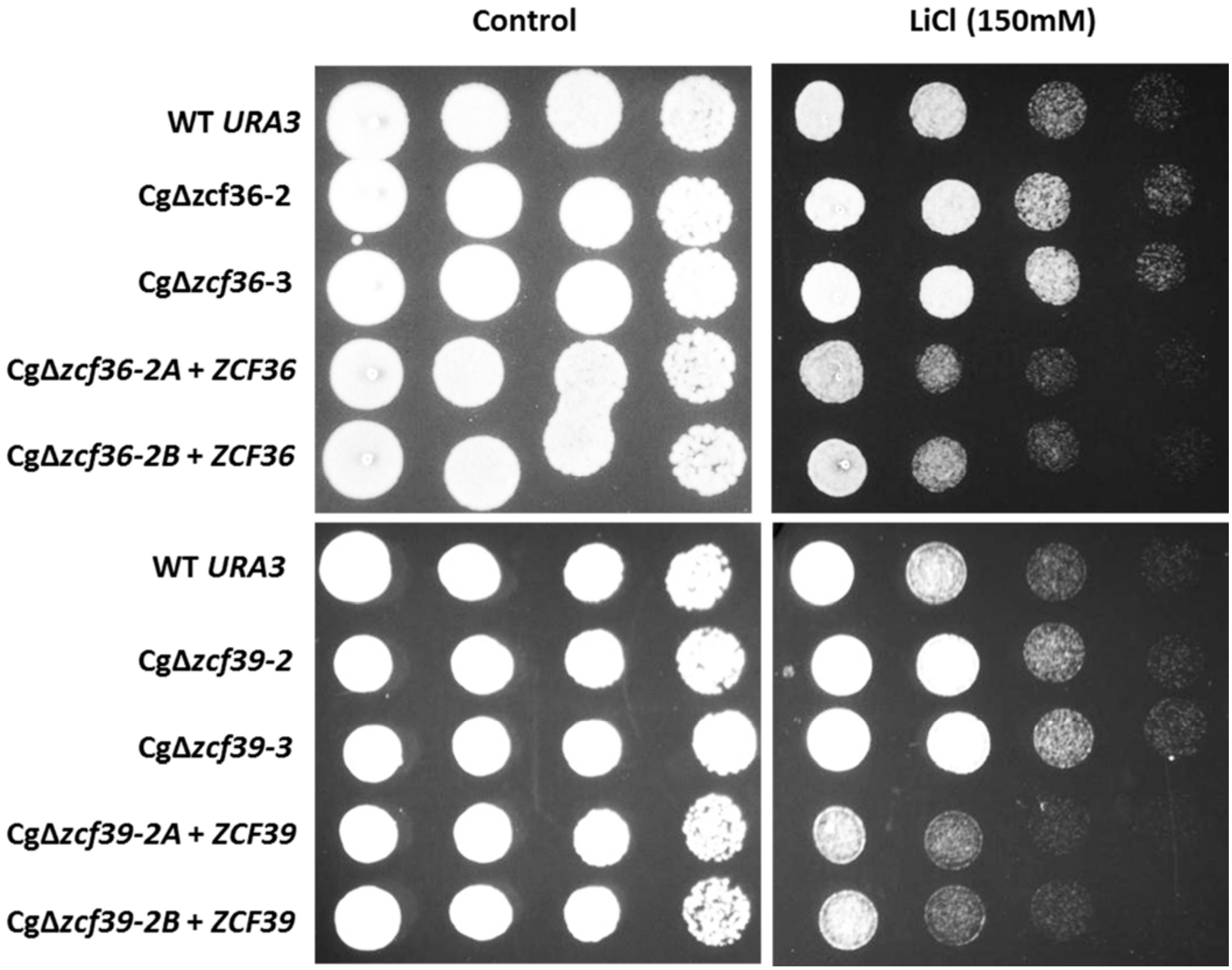
Control
LiCl (150mM)
WT URA3
CgΔzcf36-2
CgΔzcf36-3
CgΔzcf36-2A + ZCF36
CgΔzcf36-2B + ZCF36
WT URA3
CgΔzcf39-2
CgΔzcf39-3
CgΔzcf39-2A + ZCF39
CgΔzcf39-2B + ZCF39

Sensitivity to ketoconazole

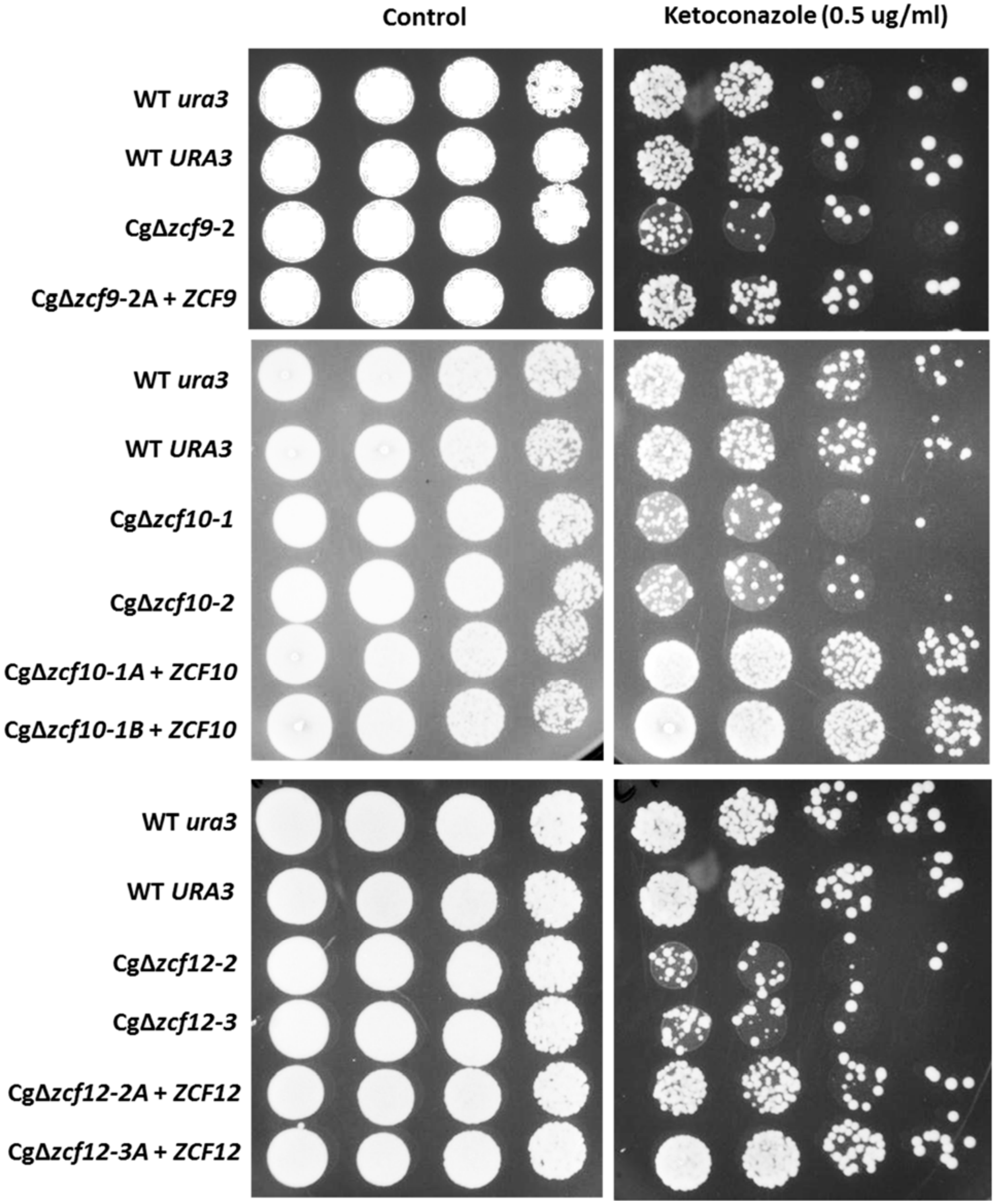

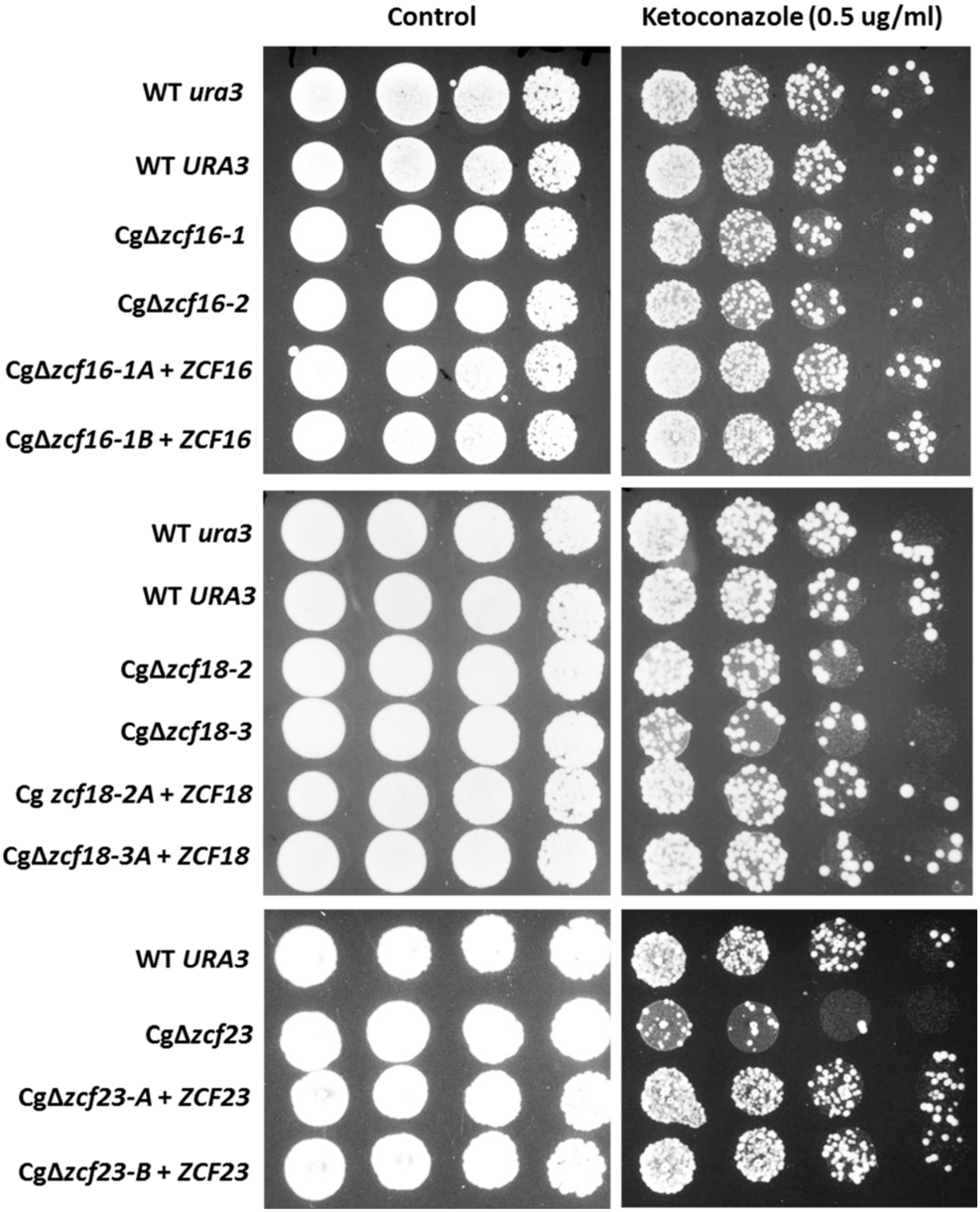
Control
Ketoconazole (0.5 ug/ml)
WT ura3
WT URA3
CgΔzcf16-1
CgΔzcf16-2
CgΔzcf16-1A + ZCF16
CgΔzcf16-1B + ZCF16
WT ura3
WT URA3
CgΔzcf18-2
CgΔzcf18-3
Cg zcf18-2A + ZCF18
CgΔzcf18-3A + ZCF18
WT URA3
CgΔzcf23
CgΔzcf23-A + ZCF23
CgΔzcf23-B + ZCF23

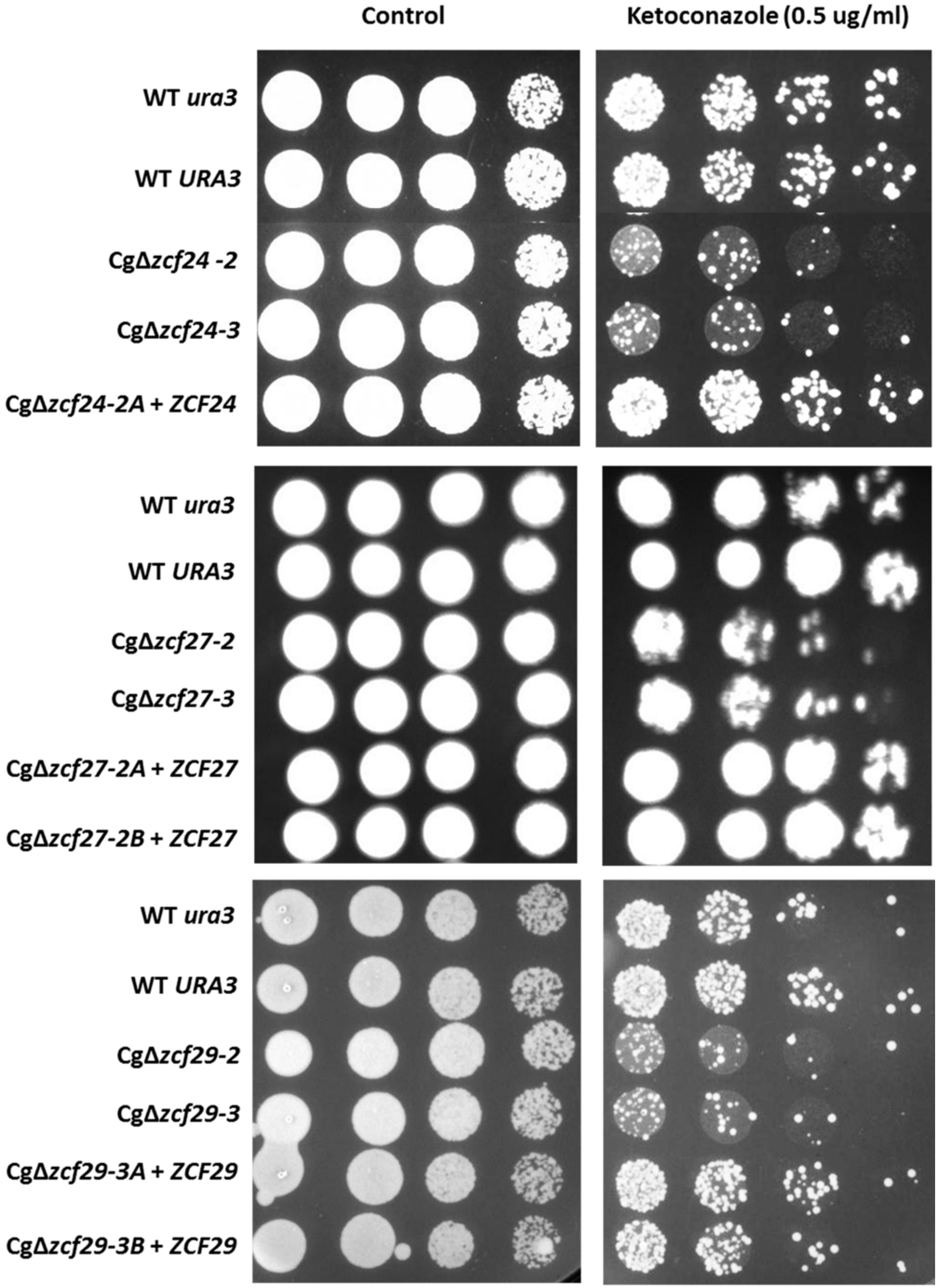
Control
Ketoconazole (0.5 ug/ml)
WT ura3
WT URA3
CgΔzcf24 -2
CgΔzcf24-3
CgΔzcf24-2A + ZCF24
WT ura3
WT URA3
CgΔzcf27-2
CgΔzcf27-3
CgΔzcf27-2A + ZCF27
CgΔzcf27-2B + ZCF27
WT ura3
WT URA3
CgΔzcf29-2
CgΔzcf29-3
CgΔzcf29-3A + ZCF29
CgΔzcf29-3B + ZCF29

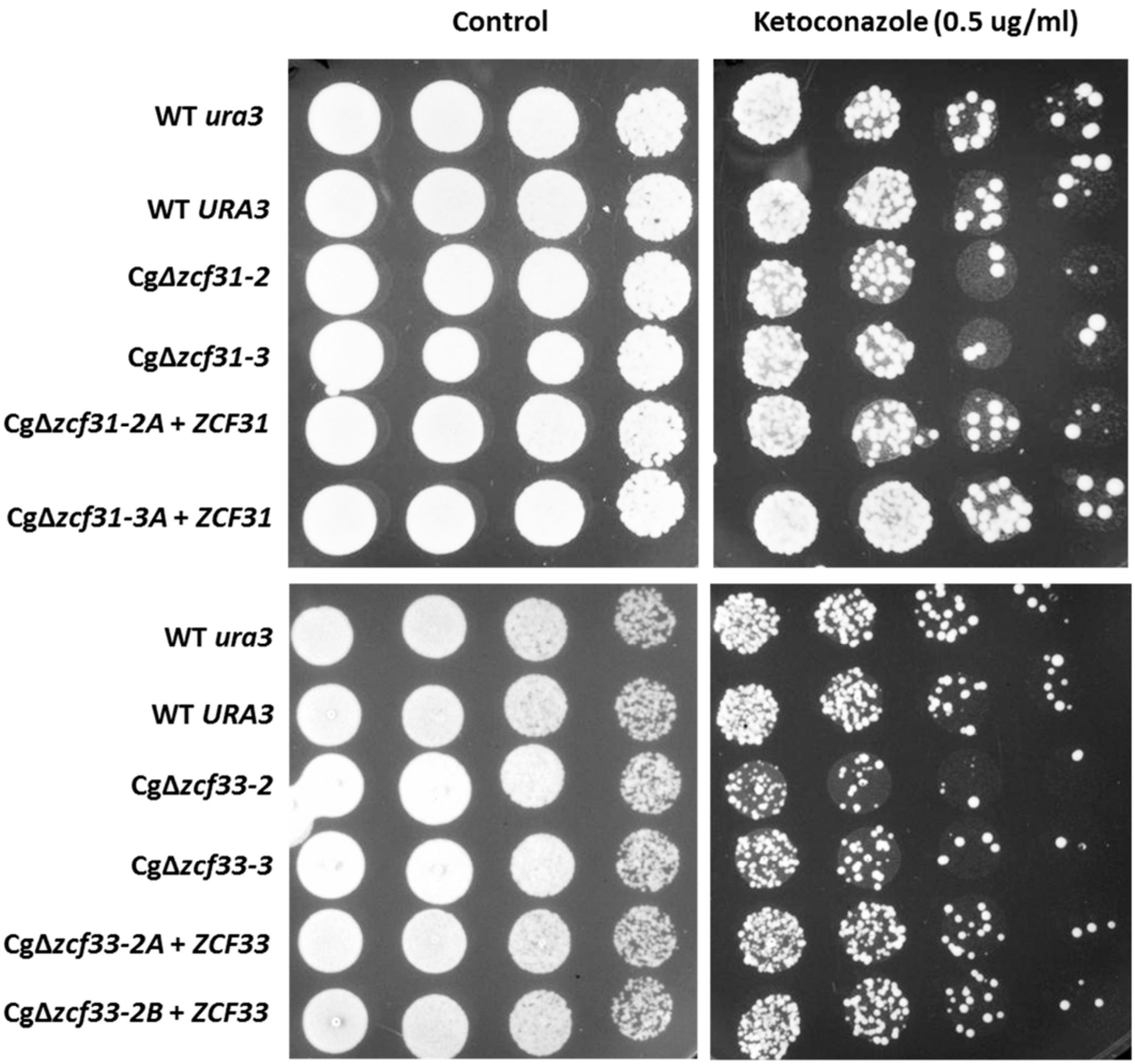

Control
Ketoconazole (0.5 ug/ml)
WT *ura3*
WT *URA3*
CgΔ*zcf31-2*
CgΔ*zcf31-3*
CgΔ*zcf31-2A* + *ZCF31*
CgΔ*zcf31-3A* + *ZCF31*
WT *ura3*
WT *URA3*
CgΔ*zcf33-2*
CgΔ*zcf33-3*
CgΔ*zcf33-2A* + *ZCF33*
CgΔ*zcf33-2B* + *ZCF33*

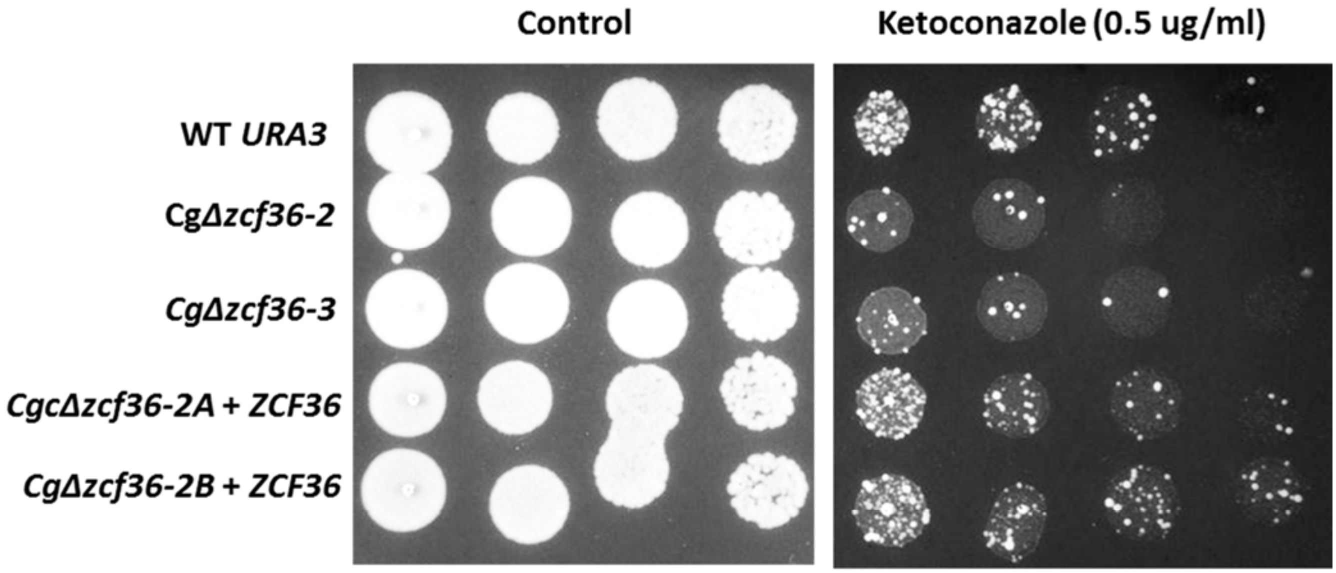

Sensitivity to SDS

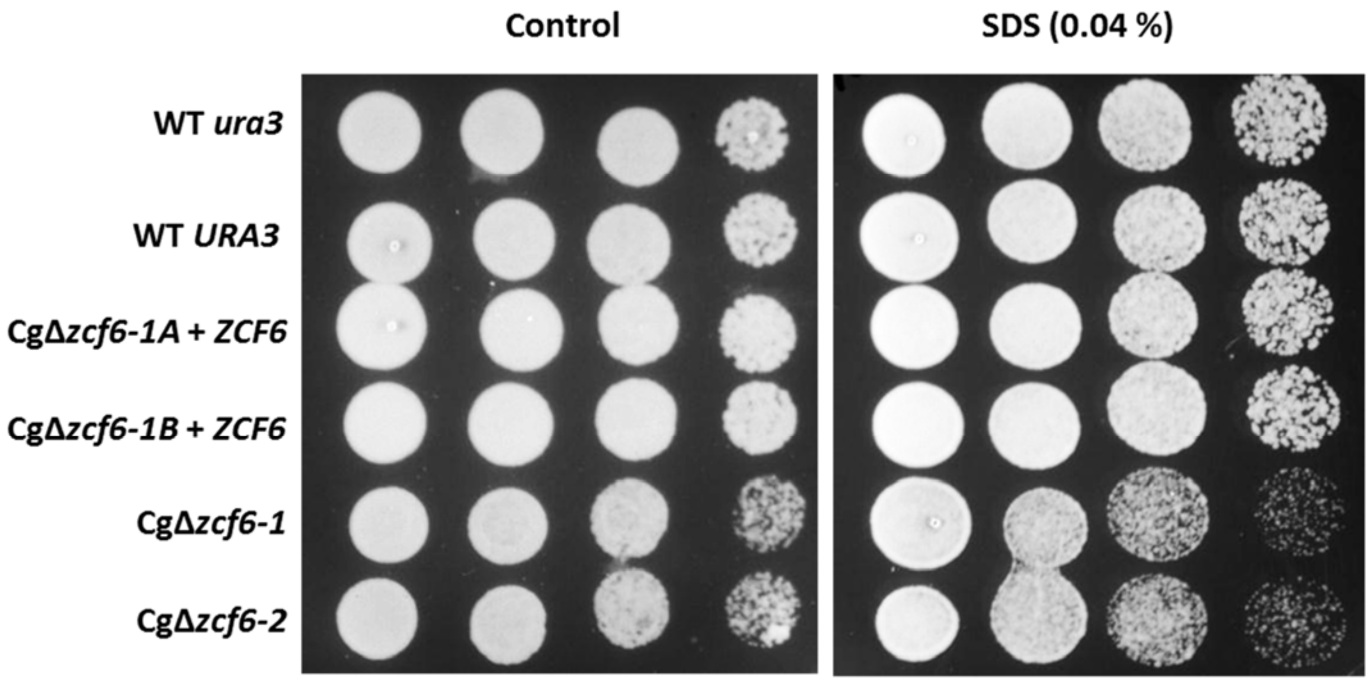

Figure S1 Additional phenotypes for strains carrying deletions of zinc cluster genes. Strains were grown overnight in rich medium, serially diluted and spotted on plates as described in Material and Methods. All deletion strains a*re* Ura^{+}. "CgΔ*zcfxx* + *ZCFXX*" are deletion strains where a wild-type allele for the zinc cluster gene *XX* has been reintroduced for complementation assays. CgΔ*zcfxx* + *ZCFXX* strains are Ura^{-}. Page 2 SI: Altered tolerance to salt (150mM LiCl). Page 5 SI: Susceptibility to ketoconazole. Page 9 SI: Sensitivity to SDS (0.04%).
